# The Retromer Coat Complex Coordinates Endosomal Sorting and Dynein-Mediated Transport, with Carrier Recognition by the *trans*-Golgi Network

**DOI:** 10.1016/j.devcel.2009.04.016

**Published:** 2009-07-21

**Authors:** Thomas Wassmer, Naomi Attar, Martin Harterink, Jan R.T. van Weering, Colin J. Traer, Jacqueline Oakley, Bruno Goud, David J. Stephens, Paul Verkade, Hendrik C. Korswagen, Peter J. Cullen

**Affiliations:** 1 The Henry Wellcome Integrated Signalling Laboratories, Department of Biochemistry, School of Medical Sciences, University of Bristol, Bristol BS8 1TD, UK; 2 Hubrecht Institute, Royal Netherlands Academy of Arts and Sciences and University Medical Center Utrecht, Uppsalalaan 8, 3584 CT, Utrecht, The Netherlands; 3 Department of Cell Biology, Institut Curie, 26, rue d'Ulm 75248, Paris cedex 05, France; 4 Department of Biochemistry, School of Medical Sciences, University of Bristol, Bristol BS8 1TD, UK; 5 Wolfson BioImaging Facility, School of Medical Sciences, University of Bristol, Bristol BS8 1TD, UK; 6 Department of Biochemistry and Department of Physiology and Pharmacology, School of Medical Sciences, University of Bristol, Bristol BS8 1TD, UK

**Keywords:** CELLBIO

## Abstract

Early endosome-to-*trans*-Golgi network (TGN) transport is organized by the retromer complex. Consisting of cargo-selective and membrane-bound subcomplexes, retromer coordinates sorting with membrane deformation and carrier formation. Here, we describe four mammalian retromers whose membrane-bound subcomplexes contain specific combinations of the sorting nexins (SNX), SNX1, SNX2, SNX5, and SNX6. We establish that retromer requires a dynamic spatial organization of the endosomal network, which is regulated through association of SNX5/SNX6 with the p150^glued^ component of dynactin, an activator of the minus-end directed microtubule motor dynein; an association further defined through genetic studies in *C. elegans*. Finally, we also establish that the spatial organization of the retromer pathway is mediated through the association of SNX1 with the proposed TGN-localized tether Rab6-interacting protein-1. These interactions describe fundamental steps in retromer-mediated transport and establish that the spatial organization of the retromer network is a critical element required for efficient retromer-mediated sorting.

## Introduction

The retromer is an evolutionarily conserved protein complex that functions to sort cargo from early endosomes back to the trans-Golgi network (TGN) (Bonifacino and Hurley, 2008; Seaman, 2005). In yeast, the retromer is composed of a cargo selective subcomplex comprizing Vps26p, Vps29p and Vps35p, and a membrane interacting subcomplex formed by Vps5p and Vps17p (Bonifacino and Hurley, 2008; Horazdovsky et al., 1997; Seaman, 2005; Seaman et al., 1998). The latter two proteins are sorting nexins (SNX), a protein family defined by the presence of a phosphoinositide-binding SNX-phox homology (SNX-PX) domain (Carlton et al., 2005; Cullen, 2008; Seet and Hong, 2006; Teasdale et al., 2001).

For the mammalian retromer, while the cargo-selective subcomplex is similar to the one in yeast (Bonifacino and Hurley, 2008), the composition of the membrane-associated SNX subcomplex is more complex and less well understood (Cullen, 2008). RNAi experiments and knockout studies in mice have established that sorting nexin-1 (SNX1) and the closely related sorting nexin-2 (SNX2) are both required for retromer-mediated endosome-to-TGN transport (Carlton et al., 2004; Griffin et al., 2005; Rojas et al., 2007). More recently, an RNAi loss-of-function screen of 30 human SNXs identified sorting nexin-5 (SNX5) and sorting nexin-6 (SNX6) as additional retromer components (Wassmer et al., 2007).

All of these mammalian retromer SNXs contain, in addition to the SNX-PX domain, a membrane-binding carboxy-terminal BAR (Bin/amphiphysin/Rvs) domain (Carlton et al., 2004). This is a dimerization motif that forms a rigid curved structure, the concave face of which constitutes a membrane-binding surface, which preferentially interacts with membranes of positive curvature as found on small vesicles or narrow-diameter membrane tubules (McMahon and Gallop, 2005). In some instances, for example, SNX1 (Carlton et al., 2004), BAR-domain-containing proteins can induce the formation of high curvature membrane tubules, possibly through the formation of a polymerized helical coat (Frost et al., 2008; McMahon and Gallop, 2005). Indeed, the mammalian retromer SNX complex generates and defines a tubular subdomain of the early endosome (Carlton et al., 2004; Rojas et al., 2007; Wassmer et al., 2007). This coat acts as a docking site for the cargo-selective VPS26-VPS29-VPS35 subcomplex, which through the binding of VPS35 to a specific sorting signal partitions retromer cargo into the SNX subdomain (Seaman, 2007). Thus, retromer couples membrane deformation with the process of cargo sorting.

Interest in retromer has recently intensified as a result of evidence linking it to a diverse array of physiological processes (Bonifacino and Hurley, 2008). These include late-stage-onset Alzheimer's disease, where retromer regulates the endosomal sorting of the β-amyloid precursor protein (APP) and its processing enzymes β- and γ-secretase, and the role of retromer in the endosomal sorting and surface delivery of Wntless, a sorting receptor for the Wnt family of morphogens, thereby establishing Wnt gradients during development (Bonifacino and Hurley, 2008). Understanding the mechanistic details of how retromer regulates endosomal sorting is therefore of significant interest.

While some of the functional components that make up the retromer are now known, hardly anything is known about the interplay between the different mammalian retromer SNXs, and virtually nothing about the mechanistic details of how retromer-mediated exit from the endosomal system is linked to the actual transport, tethering, and fusion of the cargo-enriched carriers with their target membrane. Here we provide answers to some of these fundamental questions by describing interactions between the retromer SNXs that define the existence of four mammalian retromers. In addition, we establish that efficient retromer sorting requires a dynamic spatial organization of the endosomal network that is regulated through the association of SNX5 and SNX6 with the dynactin component p150^glued^, an activator of the minus-end-directed microtubule motor dynein, and the binding of SNX1 with the proposed TGN-localized tether Rab6-interacting protein 1.

## Results

### Defining the Interaction Map between Components of the SNX-BAR Retromer Subcomplex

**Figure 1:**
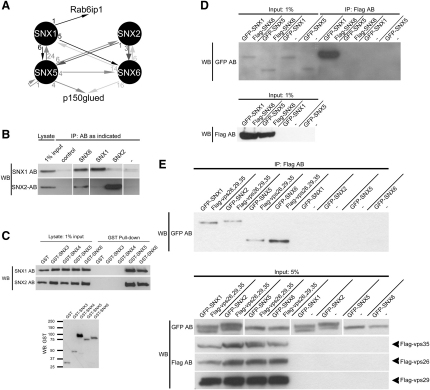
SNX1, SNX2, SNX5, and SNX6 Form a Network of Interactions (A) Results of four genome-wide yeast two-hybrid screens using SNX1, SNX2, SNX5, and SNX6 as baits. Arrows indicate interactions identified in a given screen; numbers indicate the number of hits/clones isolated. (B) Immunoprecipitation of endogenous protein by antibodies directed against either a nonrelated control (anti-sulfotransferase), SNX6, SNX1, SNX2, or omission of antibody (−) and probing for the coprecipitation of either endogenous SNX1 or SNX2. Under conditions in which SNX1 and SNX2 bind the positive control SNX6, they do not show any association with one another. (C) GST pull-downs from HeLa cells expressing either GST or GST-tagged SNX3, SNX4, SNX5, or SNX6. Endogenous SNX1 and SNX2 coprecipitate with GST-SNX5 or GST-SNX6 but not with any of the controls. The anti-GST blot shows expression levels of the GST-fusions. (D) Immunoprecipitation of Flag-SNX6 from HeLa cells coexpressing either GFP-SNX1 or GFP-SNX5 demonstrates that while the positive control GFP-SNX1 strongly coprecipitates with Flag-SNX6, GFP-SNX5 does not. (E) Association of SNX1, SNX2, SNX5, or SNX6 with the retromer cargo-selective subcomplex formed by VPS26, VPS29, and VPS35 was analyzed by immunoprecipitation. Flag-VPS26, VPS29, and VPS35 were coexpressed with GFP-SNX1, GFP-SNX2, GFP-SNX5 or GFP-SNX6, in HEK293T cells prior to immunoprecipitation using immobilized Flag antibody. Control cells expressed the GFP-fusion alone.

To identify molecular interactions important in regulating retromer function, we performed a series of yeast two-hybrid screens against a human brain library using as bait the individual SNX-BAR proteins that have been functionally proposed to form the mammalian-membrane-bound subcomplex (Carlton et al., 2004; Rojas et al., 2007; Wassmer et al., 2007). From these screens, a total of 130 clones were verified as true positives as determined by back screens (see Table S1 available online). One outcome from the sequencing of these clones was the observation of a network of interactions between the individual retromer SNX-BARs (Figure 1A). These appeared to be specific, as none of the other 29 mammalian SNXs, or any other BAR-domain-containing proteins, were isolated (Table S1). When using SNX1 as bait, clones encoding SNX5 and SNX6 were identified; the corresponding SNX5-SNX1 and SNX6-SNX1 interactions were also identified in screens using as baits SNX5 or SNX6 (Figure 1A). Similarly, when using SNX2 as bait, associations with SNX5 and SNX6 were identified; again, these associations were observed when using SNX5 or SNX6 as baits (Figure 1A). Interestingly, no association between SNX1 and SNX2 was observed, and interactions between SNX5 and SNX6 were only observed in the screen using SNX5 as bait (Figure 1A). Furthermore, self-self interactions were not observed, with one exception: a single SNX5 clone being isolated from the SNX5 screen (Figure 1A; Table S1).

To verify these interactions, we performed immunoprecipitations from HeLa cell lysates, using either antibodies that recognize endogenous protein for SNX1 and SNX2 (Carlton et al., 2004; Carlton et al., 2005) or, as no antibodies were available that were specific enough to discriminate between the closely related proteins SNX5 and SNX6 (∼65% identical), transfected tagged constructs. Under conditions in which SNX1 and SNX2 both associated with SNX6, no association between SNX1 and SNX2 was observed (Figure 1B). Confirming further the interactions observed from the two-hybrid screens, SNX1 and SNX2 both associated with GST-tagged SNX5 and SNX6 (Figure 1C). In contrast, while interactions between SNX5 and SNX6 were observed in the two-hybrid screen (Figure 1A), when Flag-tagged SNX6 was expressed together with GFP-tagged SNX5, while SNX6 strongly associated with SNX1 as a positive control, no association with SNX5 was detectable (Figure 1D). These associations were specific, as no interaction was observed between either endogenous SNX1 or SNX2 and SNX3 (a non-SNX-BAR protein) or SNX4 (a SNX-BAR protein not implicated in retromer biology) (Figure 1C).

These data establish that for those SNX-BARs that functionally define the retromer membrane-bound subcomplex, two groupings can be observed: one formed by SNX1 and SNX2, another by SNX5 and SNX6. While SNXs of one group interact with SNXs of the other group, under the same conditions, interactions of SNXs within a group are biochemically not observed.

### Phylogenetic Analysis of Retromer Sorting Nexins

To better understand the relationship between these four mammalian SNX-BARs, a phylogenetic tree was constructed (Figure S1A), including the retromer sorting nexins of *S. cerevisiae*, *C. elegans, D. melanogaster, S. purpuratus, D. Rerio, X. laevis,* mouse, and humans. The phylogenetic tree showed that a duplication of the retromer sorting nexins has occurred between the invertebrate sea urchin *Strongylocentrotus purpuratus* and the vertebrate *Danio rerio*, with the vertebrate gene pair SNX1/SNX2 arising from one ancestral gene and the SNX5/SNX6 pair from another. Unfortunately, the sequence conservation between the yeast gene pair Vps5/Vps17 and the vertebrate SNX1/SNX2/SNX5/SNX6 are not sufficient to deduce with certainty whether SNX1/SNX2 are orthologs of Vps5 or whether SNX5/SNX6 are orthologs of Vps17. The more similar domain structures of these proteins might, however, suggest this to be the case (Figure S1B).

### SNX1, SNX2, SNX5, and SNX6 Associate with the Cargo Selective Retromer Subcomplex

Previously, only the association of SNX1 with the cargo-selective subcomplex had been documented (Gullapalli et al., 2004). We therefore addressed whether the other retromer SNX-BARs could associate with this subcomplex. Flag-tagged VPS26, VPS29, and VPS35 were coexpressed in HEK293T cells together with GFP-tagged SNX1, SNX2, SNX5, or SNX6. Using immobilized Flag antibody, precipitation of the cargo-selective subcomplex from cell lysates revealed that each GFP-tagged SNX-BAR was coimmunoprecipitated (Figure 1E). These biochemical data, together with the association of the SNX1/SNX2 and SNX5/SNX6 pairs with each other, co-localization experiments of all retromer SNXs shown in Figure S2 and the functional data provided previously (Carlton et al., 2004; Griffin et al., 2005; Rojas et al., 2007; Wassmer et al., 2007), establish that these SNX-BARs are functional components of the mammalian retromer.

### SNX5 and SNX6 Associate with the p150^glued^ Subunit of Dynactin

**Figure 2:**
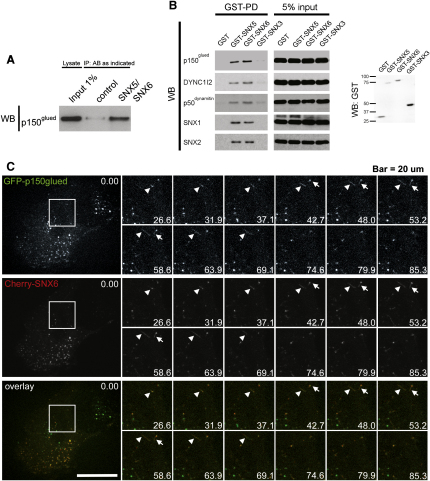
SNX5 and SNX6 Associate with p150^glued^ (A) Coimmunoprecipitation of endogenous p150^glued^ from HeLa cell lysates with SNX5 and SNX6 antibodies demonstrates the association of p150^glued^ with SNX5/SNX6 in vivo. To maximize the efficiency of the IP, a combination of two different antibodies was used. (B) GST pull-down of endogenous p150^glued^, DYNC1I2, p50^dynamitin^, SNX1 and SNX2 from HEK293T cells expressing either GST-SNX5, GST-SNX6, GST-SNX3, or GST (expression level of GST-fusions shown by the anti-GST blot). While both GST-SNX5 and GST-SNX6 coprecipitate p150^glued^, DYNC1I2, p50^dynamitin^, SNX1 and SNX2, GST-SNX3, and GST do not. (C) Stills of Movie S1 at various time points, displaying a HeLa cell expressing lentivirally transduced Cherry-SNX6 and GFP-p150^glued^. Colocalization and comovement of retromer-positive vesicles and tubules decorated with p150^glued^ are indicated by the arrow and the arrowhead in the insets. Bar, 20 μm.

The two-hybrid screens of SNX5 and SNX6 also identified interactions with the C-terminal region of p150^glued^ (DCTN1): no interactions were observed with SNX1 or SNX2 (Figures 1A and Figure S3). p150^glued^ is a subunit of dynactin that activates the minus-end-directed motor dynein (Schroer, 2004; Vaughan and Vallee, 1995). To verify these interactions, we immunoprecipitated endogenous SNX5 and SNX6 from HeLa cell lysates and probed for coprecipitation of endogenous p150^glued^ (Figure 2A). p150^glued^ was enriched in dual SNX5/SNX6 immunoprecipitates when compared with control levels (Figure 2A). Additionally, when GST-SNX5 and GST-SNX6 were expressed in HEK293T cells, isolation of associating proteins by GST affinity chromatograph revealed the presence of not only endogenous p150^glued^ but also another component of the multisubunit dynactin complex, p50^dynamitin^, and, importantly, the dynein intermediate chain (DYNC1I2) (Figure 2B). Attempts at mapping the interaction site for p150^glued^ on SNX6 proved unsuccessful, as even minor truncation of this sorting nexin led to its destablization when expressed in HEK293T or HeLa cells (data not shown). Overall, these data are therefore consistent with retromer existing in a complex with the dynein-dynactin motor through the binding of SNX5 and SNX6 to p150^glued^.

Further evidence for the association of p150^glued^ with these SNX-BARs came from live-cell confocal imaging. When coexpressed, mCherry-SNX6 colocalized with GFP-p150^glued^, expressed at low levels, on numerous vesicular structures and tubules where they underwent comovement (Figure 2C and Movie S1). Similar results were obtained for SNX5 (data not shown). Thus, in vivo p150^glued^ is associated with retromer SNX-decorated endosomes and tubules.

### RNAi-Mediated Suppression of p150^glued^ Perturbs Retromer Function

**Figure 3:**
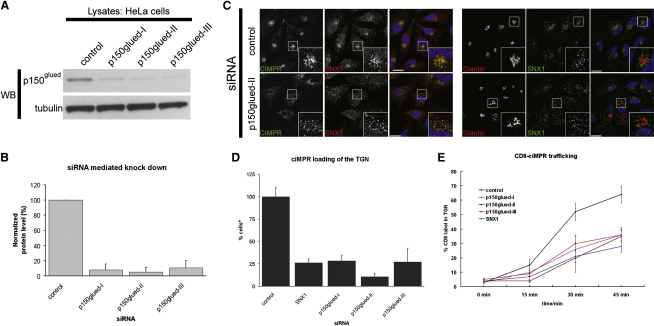
Suppression of p150^glued^ Expression Perturbs the Efficiency of Retromer-Mediated Endosome-to-TGN Transport (A) Demonstration of knock down of p150^glued^ in HeLa cells using three different siRNAs and probing for endogenous p150^glued^, using tubulin staining to indicate equal loading. (B) Quantification of knock down obtained in four different experiments for p150^glued^ suppression using three different siRNAs each. Error bars indicate standard deviation. (C) Suppression of p150^glued^ affects the steady-state distribution of CI-MPR in HeLa cells, leading to a peripheral dispersal of the label in comparison to control cells, phenocopying loss of retromer components. Note that under these conditions, knock down of p150^glued^ does not affect the general organization of the giantin-stained Golgi, the TGN, the microtubule array, or EEA1-positive early endosomes, while a minor alteration in the distribution of the CD63-positive late endosomal/lysosomal compartment is apparent (Figure S4). (D) Quantification of the number of Golgis with more than half-maximal intensity of CI-MPR staining expressed as percent of total Golgis analyzed, using three different siRNAs for p150^glued^ suppression and including SNX1 knock down as a positive control for defective retromer trafficking. Error bars indicate standard deviation. (E) Kinetics of the uptake of a CD8-tagged CI-MPR from the plasma membrane and trafficking to the TGN, quantified as percent of CD8 label in the TGN. Knock down of p150^glued^ or SNX1 induces a major reduction in the efficiency of transport to the TGN in comparison to control cells. Error bars indicate standard deviation.

To establish the physiological relevance of this association, we combined RNAi-mediated suppression with functional, retromer-dependent endosome-to-TGN transport assays. Three independent siRNAs, each targeting different regions of the p150^glued^ message, were transiently transfected into HeLa cells, and the levels of knock down obtained were quantified by western blots (Figures 3A and 3B). To assay retromer function in a p150^glued^ suppressed background, we examined by confocal microscopy the steady-state distribution of endogenous CI-MPR, an established retromer cargo (Arighi et al., 2004; Carlton et al., 2004; Seaman, 2004). Under these conditions, the CI-MPR was redistributed from its normal steady-state TGN localization into peripherally dispersed punctae, where it colocalized with markers of the retromer-positive endosome (Figure 3C). This phenotype was also observed in HeLa cells suppressed for SNX1 and has previously been established to arise from a defect in retromer-mediated endosome-to-TGN transport (Arighi et al., 2004; Carlton et al., 2004; Seaman, 2004). To quantify this, we calculated the percentage of cells with more than half maximum CI-MPR loading of the Golgi apparatus as defined by giantin staining (Figure 3D). This revealed a decrease when either p150^glued^ or SNX1 were suppressed. Depletion of p150^glued^ did not induce a gross alteration in the morphology of the Golgi apparatus (Figure 3C and Figure S4), thereby excluding the possibility that the CI-MPR phenotype arose from a general, nonspecific perturbation in Golgi function.

Another method of probing retromer function is to define the kinetics of transport of a CD8-tagged CI-MPR as it undergoes endocytosis from the cell surface and sorting through endosomes en route to the TGN (Carlton et al., 2004; Seaman, 2004; Wassmer et al., 2007) (Figure S5 and Movie S2). By measuring the colocalization of the CD8 label with the TGN marker TGN46, we observed that the endosomal sorting of the CD8-CI-MPR to the TGN was strongly defective when p150^glued^ was suppressed: half maximal CD8-CI-MPR loading at 22 min was reduced from 32% in control cells to between 10% (p150^glued^-II siRNA) and 18% (p150^glued^-III siRNA) in p150^glued^ suppressed cells (Figure 3E). The association of p150^glued^ is therefore required for efficient retromer-mediated endosome-to-TGN transport.

### p150^glued^ Is Required for the Spatial Organization of the Retromer-Labeled Endosomal Network

**Figure 4:**
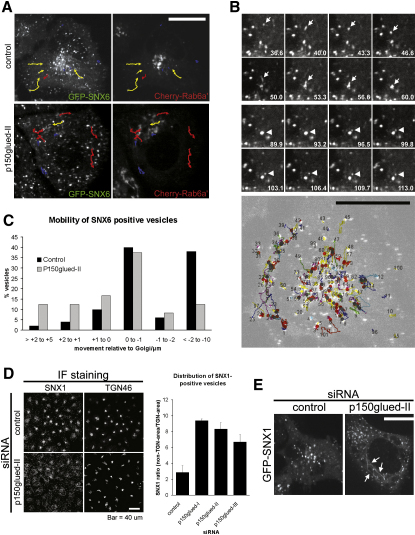
Association with p150^glued^ Regulates the Long-Range Transport and Steady-State Distribution of SNX6-Labeled Endosomes (A) Live-cell confocal imaging of HeLa cells lentivirally transduced with GFP-SNX6 and mCherry-Rab6a′ and then treated with control or p150^glued^ siRNA. Selected single cells are shown corresponding to the initial frame of Movies S3 and S5. The tracks of selected GFP-SNX6-labeled endosomes are marked with arrows indicating the direction of movement over time. Yellow arrows indicate movement toward the TGN (defined by mCherry-Rab6a′), red arrows movement away from the TGN, and blue traces little net movement over the duration of the imaging. Note that the distribution of GFP-SNX6 structures is altered between the control and p150^glued^-suppressed cell. Bar, 20 μm. (B) Imaging sequence of vesicles in a control cell illustrates what appears to be a fusion event between two GFP-SNX6-positive structures (arrow) and subsequently a fission event (arrowhead) from the same structure. Time in seconds from the beginning of Movie S4 is indicated. Reconstruction of movements of GFP-SNX6-positive structures over 5 min in a HeLa cell illustrates the complexity of the retromer sorting system. Starting points of vesicles are indicated by numbers, visible merging events that most likely represent fusion events by a red dot, splitting of structures by gray spots. Note the occurrence of several merging and splitting events on most traces that travel notable distances. Bar, 20 μm. (C) Knock down of p150^glued^ affects mobility of GFP-SNX6-positive carriers relative to the TGN. About 50 vesicles per condition were tracked over 5 min and the distance traveled toward the Golgi between the start and the end point measured. Percent of vesicles per distance class are plotted in the histogram, with positive values indicating net movement away from the Golgi and negative values indicating movement toward the Golgi. Knock down of p150^glued^ shifts the distribution toward greater distances from the Golgi at the endpoint of the movie and strongly reduces the group of about 38% of carriers that move far distances toward the Golgi in control cells. (D) Imaging and quantification of the redistribution of vesicles decorated with endogenous SNX1 from the Golgi area toward the cell periphery by suppression of p150^glued^. Bar, 40 μm. (E) Suppression of p150^glued^ leads to the occurrence of long and relatively static SNX1-decorated tubules in a subset of cells (∼20%) from a clonal HeLa cell line expressing GFP-SNX1. Bar, 20 μm.

It is well established that dynein is an important motor complex in determining the localization and movement of a wide array of organelles, including endosomes (Driskell et al., 2007; Johansson et al., 2007; Jordens et al., 2001). Our characterization of the indirect coupling of retromer to dynein is consistent with this minus-end-directed microtubule motor playing a role in the transport of retromer-decorated endosomes and carriers toward the juxtanuclear TGN, this organelle being close to the microtubule-organizing center (MTOC). Indeed, it has previously been established that in HeLa cells, retromer-labeled endosomes have their greatest density in this juxtanuclear region (Carlton et al., 2004; Seaman, 2004) (Figure 3C). In live-cell imaging of lentivirally transduced GFP-SNX6, individual SNX6-positive structures were observed to undergo stochastic short- and long-range movement from the cell periphery both toward and away from the TGN (Figure 4A and Movie S3). Dual live-cell imaging of mCherry-SNX6 with the plus-end microtubule tracking protein GFP-EB3 revealed that movement toward the cell center was minus-end directed, consistent with the coupling of retromer to the dynein-dynactin motor complex (Figure S6). Interestingly, during the course of movement from the cell periphery, SNX6-labeled vesicles underwent several fission and fusion events with other SNX6-positive structures as they tracked toward the TGN (Figure 4B and Movie S4).

In p150^glued^-suppressed cells, the steady-state enrichment of structures labeled for GFP-SNX6 or endogenous SNX1 around the TGN was lost and a dispersed peripheral localization observed (Figures 4A and 4D and Movie S5). We analyzed the tracking of individual GFP-SNX6-labeled vesicles over a period of 5 min, imaging at a frame approximately every 3 s. In control cells (Figure 4C), three distinct populations of vesicles movements could be resolved: one population (40%) moved relatively short distances toward the TGN, between 0 and 1 μm, while another (44%) tracked greater than 1 μm toward this region with a subpopulation (38%) that moved between 2 and 10 μm, and, finally, a small population (16%) actually moved away from the TGN (Figure 4C). In p150^glued^ suppressed cells the distribution of these populations was altered such that while the population of vesicles that moved relatively short distances to the TGN appeared unaffected (37%), the frequency of those vesicles tracking greater than 1 micron decreased. Indeed, the sub-population of these vesicles that tracked between 2 and 10 microns fell to just 12%. This decrease in movement toward the TGN was coupled with an increase in the frequency of vesicles moving away from the TGN (Figure 4C). The combination of a diminished pool of long-range vesicle movements toward the TGN and an increase of movement away from the TGN in p150^glued^ suppressed cells establishes that the association with p150^glued^ is crucial for enabling retromer decorated carriers to move toward the TGN. Moreover, this movement is required to organize the steady-state spatial distribution of the retromer-labeled endosomes to regions surrounding the TGN, which in itself would appear to be required for efficient retromer-mediated sorting.

Finally, in a minor population of p150^glued^-suppressed cells (approximately 18%) from a clonal HeLa cell line stably expressing GFP-SNX1, we observed the appearance of extended SNX1-decorated tubules (Figure 4E). This phenotype was not detectable in cells transfected with control siRNA, indicating that the p150^glued^-mediated association of retromer-labeled tubules with the dynein-dynactin motor complex might be necessary for processing of the tubules, most likely by applying longitudinal force that assists the fission of tubules from the endosomal membrane, a yet ill-defined event.

### *C. elegans* p150^glued^/DNC-1 Is Required for EGL-20/Wnt Signaling

Further evidence for a function of p150^glued^ in retromer-dependent trafficking is provided by the identification of the *C. elegans* p150^glued^ ortholog *dnc-1* in a genome-wide RNAi screen for genes that are required for signaling by the Wnt protein EGL-20 (M.H. and H.C.K., data not shown).

**Figure 5:**
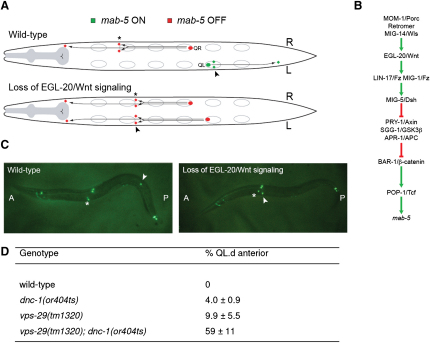
p150^glued^/DNC-1 Is Required for EGL-20/Wnt Signaling in *C. elegans* (A) Schematic representation of the migration of the left and right Q neuroblast descendants (dorsal view). On the left side (“L”), the Q descendants express the homeobox gene *mab-5* (green) and migrate toward the posterior. On the right side (“R”), the Q descendants do not express *mab-5* (red) and migrate in the default anterior direction. Mutations that disrupt EGL-20 production, secretion, or signaling lead to a loss of *mab-5* expression in the left Q neuroblast and to the anterior migration of its descendants. (B) EGL-20/Wnt triggers a canonical Wnt/β-catenin signaling pathway. (C) Visualization of the Q descendants using a *mec-7::gfp* reporter that is expressed in the left Q descendant PVM (arrow head), the right Q descendant AVM (asterisk)m and four other neurons. In wild-type animals, AVM is in an anterior position and PVM in a posterior position. In mutants that disrupt EGL-20 signaling, both AVM and PVM are in the anterior. (D) Synchronized embryos were shifted to the restrictive temperature of 25°C and grown for 2 days; the final positions of the left Q descendants (QL.d) were scored using the *mec-7::gfp*-expressing transgene *muIs32* (Ch'ng et al., 2003). n > 400 out of two independent experiments. Data are represented as mean ± SD.

It has recently been shown that secretion of Wnt proteins is mediated by the seven-pass trans-membrane protein Wntless (Wls) (Banziger et al., 2006; Bartscherer et al., 2006). A prerequisite for efficient Wnt secretion is the recycling of plasma-membrane-localized Wls back to the TGN through a retromer-dependent trafficking pathway (Franch-Marro et al., 2008; Pan et al., 2008; Port et al., 2008; Yang et al., 2008). In the absence of this recycling step, Wls is degraded in lysosomes and Wnt signaling is impaired. In *C. elegans*, mutation of the retromer complex results in a strong defect in signaling by EGL-20/Wnt (Coudreuse et al., 2006). One of the functions of EGL-20/Wnt is to control the left-right asymmetric migration of the Q neuroblast descendants, and the final positions of these cells provide a sensitive assay for Wnt signaling activity (Whangbo and Kenyon, 1999) (Figures 5A–5C).

To further investigate the requirement of *dnc-1* in EGL-20/Wnt signaling and to circumvent the essential function of *dnc-1* during early development, we used the temperature-sensitive allele *dnc-1(or404ts)*, which is fully viable at the permissive temperature but shows a loss-of-function phenotype at restrictive temperatures (Encalada et al., 2005; Zhang et al., 2008). We found that shifting animals to the restrictive temperature during embryogenesis resulted in a weak but significant defect in EGL-20/Wnt signaling (as shown by the anterior localization of the left Q descendants in Figure 5D). Next, we investigated whether *dnc-1(or404ts)* enhances the EGL-20/Wnt signaling phenotype of *vps-29(tm1320)*. We have previously shown that the Wnt phenotype of *vps-29(tm1320)*, which is much weaker than that of *vps-26* or *vps-35* mutants, can be strongly enhanced by mutations in other Wnt pathway components, including hypomorphic mutations of the Wls ortholog *mig-14* (Yang et al., 2008). As shown in Figure 5D, we found that *dnc-1(or404ts)* also strongly enhances the EGL-20/Wnt phenotype of *vps-29(tm1320)*. Combined with our interaction and functional studies in mammalian cells, these results provide further support for a function of p150^glued^/DNC-1 in retromer-dependent trafficking.

### Rab6IP1 Assists p150^glued^ in Defining the Spatial Organization of the Retromer Retrieval Pathway

The association of p150^glued^ with retromer has identified the spatial lay-out of the retromer-labeled endosomal network as a key feature of efficient retromer-mediated sorting. Other components that may be important in determining such an organization are TGN-localized tethers. For example, suppression of GCC185, a TGN-localized protein required for the tethering of carriers arriving by the Rab9-dependent transport of CI-MPR from the late endosome, results in the dispersal of Rab9-positive vesicles into the cell periphery (Derby et al., 2007). We were therefore intrigued by the identification of an interaction between SNX1 and the C-terminal region (amino acids 1260–1287) of the TGN-resident protein Rab6IP1 (Figure 1A), which has been proposed to function in the tethering/docking of endosomal-transport intermediates with the TGN (Miserey-Lenkei et al., 2007).

**Figure 6:**
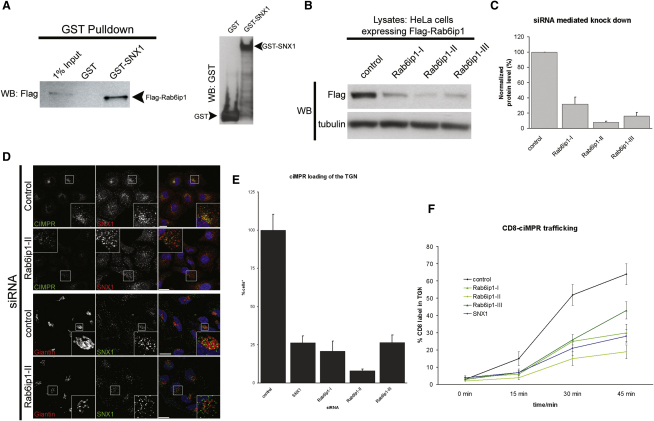
Association of SNX1 with Rab6IP1 Is Required for Retromer-Mediated Sorting (A) GST pull-down of bacterially expressed SNX1 demonstrates that GST-SNX1 can associate with FLAG-tagged Rab6IP1. Comparative levels of GST-SNX1 and GST expression are shown. (B) Knock down of Rab6IP1 using three different siRNAs and probing for Flag-Rab6IP1 lentivirally transduced into HeLa cells. (C) Quantification of Rab6IP1 knock down obtained in three different experiments using three different siRNAs. Error bars indicate standard deviation. (D) Knock down of Rab6IP1 influences the steady-state distribution of CI-MPR, leading to a peripheral dispersal of staining in comparison to control cells, phenocopying loss of retromer components. Knock down of Rab6IP1 does not appear to affect the organization of the TGN (Figure S4). (E) Quantification of the number of Golgis with more than half-maximal intensity of CI-MPR staining shows that knock down of Rab6IP1 induces a defect in CI-MPR recycling to the TGN. Error bars indicate standard deviation. (F) Kinetics of the trafficking of CD8-CI-MPR from the cell surface to the TGN shows that loss of Rab6IP1 reduces the efficiency of retromer-mediated transport in comparison to control cells. Error bars indicate standard deviation.

**Figure 7:**
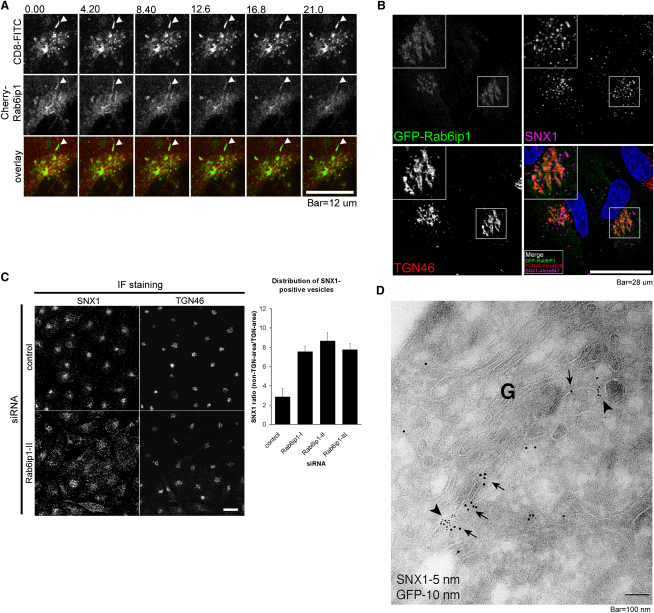
A Population of SNX1-Labeled Endosomes Resides in Close Proximity to the Rab6IP1-labeled TGN (A) Live-cell imaging shows an incoming carrier containing CD8-CI-MPR labeled with a CD8-FITC antibody colocalizing with a moving, mCherry-Rab6IP1-decorated fiber emanating from the TGN (arrowheads) (Movie S7). Time in seconds. Bar, 12 μm. (B) Triple labeling of GFP-Rab6IP1, TGN46, and SNX1 shows Rab6IP1 colocalization with the TGN, with numerous SNX1-labeled endosomes being concentrated in close proximity to TGN cisternae. Bar, 28 μm. (C) Imaging and quantification of the redistribution of vesicles decorated with endogenous SNX1 from the Golgi area towards the cell periphery by suppression of Rab6IP1. Bar, 28 μm. Error bars indicate standard deviation. (D) Electron micrograph of a Golgi/TGN complex of GFP-Rab6IP1 expressing HeLa cells immunolabeled for GFP (10 nm gold) and endogenous SNX1 (5 nm gold). SNX1 labeling is found in clusters on presumptive TGN tubulo-vesicular profiles (arrowheads). Rab6IP-1 labeling is mainly found on the same TGN profiles (arrows) and occasionally colocalizes with SNX1. Scale bar represents 100 nm.

To verify the interaction of SNX1 with Rab6IP1, GST pull-downs were performed. GST, GST-SNX1, and GST-SNX4 were recombinantly expressed in *E. coli* and purified by GST affinity chromatography. Purified protein was incubated with lysate of HeLa cells lentivirally transduced with Flag-Rab6IP1, and complexes were isolated by GST pull-downs. Under these conditions, Flag-Rab6IP1 associated with GST-SNX1 (Figure 6A) but not the control GST-SNX4 (Figure S7). Functionally, RNAi-mediated suppression of Rab6IP1 revealed that the steady-state enrichment of endogenous CI-MPR at the TGN was perturbed in Rab6IP1-suppressed cells, with the CI-MPR being dispersed into peripheral SNX1-positive endosomes (Figures 6D and 6E). Efficient suppression was achieved with three independent siRNAs (Figures 6B and 6C). The kinetics of CD8-CI-MPR transport to the TGN was strongly perturbed in Rab6IP1-suppressed cells (Figure 6F), demonstrating a functional role for Rab6IP1 in retromer-mediated endosome-to-TGN transport. To further this analysis, we examined the dynamics of CI-MPR transport to the TGN in live cells by following a CD8-CI-MPR chimera labeled with a CD8-FITC antibody (Figure S5). Incoming carriers containing CD8-CI-MPR were observed to associate with mCherry-Rab6IP1-decorated TGN membrane processes (Figure 7A and Movie S6).

To better understand the interplay between Rab6IP1 and incoming carriers, we confirmed the colocalization of GFP-Rab6IP1 with the TGN marker TGN46 (Miserey-Lenkei et al., 2007) and revealed that a population of SNX1-labeled endosomes was in close proximity to this organelle (Figure 7B). Moreover, ultrastructural analysis confirmed that SNX1-labeled endosomes were associated with the Rab6IP1-decorated TGN (Figure 7D). Importantly, suppression of Rab6IP1 resulted in a redistribution of endogenous SNX1-labeled endosomes to a more evenly scattered distribution throughout the cell, with the accumulation near to the TGN being reduced (Figure 7C). This alteration, together with the mirrored dispersal of CI-MPR (Figure 6D), is consistent with the decrease in efficiency of CI-MPR transport in Rab6IP1-suppressed cells. These data highlight further that the spatial organization of the retromer-labeled endosome is a crucial determinant of efficient retromer-mediated sorting and suggest that Rab6IP1 functions as a TGN-resident tether that, by recognizing and anchoring incoming carriers at the TGN, assists in establishing the steady-state distribution of Rab6IP1-suppressed cells.

## Discussion

Here, we have defined molecular interactions that occur between individual SNX-BAR proteins in the membrane-bound subcomplex of the mammalian retromer. In doing so, we have established that a number of retromer complexes exist, each with a distinct SNX-BAR membrane-bound subcomplex. By identifying associations with p150^glued^ and Rab6IP1, we have described molecular interactions that couple the long-range transport of retromer-labeled endosomes and carriers toward the TGN with their recognition by this recipient membrane. Importantly, these associations help to establish and maintain a steady-state enrichment of retromer-labeled endosomes and carriers to regions surrounding the TGN, a spatial organization required for efficient retromer-mediated sorting.

### The Retromer SNX-BAR Interaction Map

We have described how each SNX-BAR within two defined pairs, SNX1/SNX2 and SNX5/SNX6, can associate with a SNX-BAR encoded by the other gene pair, thereby generating four possible combinations: SNX1–SNX5, SNX1–SNX6, SNX2–SNX5, and SNX2–SNX6. Such restricted interactions conserve the binding activity of the unduplicated predecessor pair (Horazdovsky et al., 1997). Our data leads to the conclusion that SNX1/SNX2 and SNX5/SNX6 constitute the respective mammalian orthologs of yeast Vps5p and Vps17p and is therefore consistent with the idea of functional redundancy between SNX1 and SNX2 (Griffin et al., 2005; Rojas et al., 2007). While mouse genetics clearly shows that SNX1 and SNX2 are functionally redundant, at least one of them being required for embryonic survival (Griffin et al., 2005), it is less clear whether there might be tissue-specific variations in sorting nexin expression and requirement. Such a variation might explain why we previously detected a retromer-like phenotype upon SNX1, but not SNX2, suppression (Carlton et al., 2005), while a similar study from the Bonifacino laboratory found a functional requirement for both SNX1 and SNX2 in retromer-mediated sorting (Rojas et al., 2007).

As each of the four SNX-BAR combinations has an ability to associate with the cargo-selective subcomplex, there is an increase in the potential diversity of the retromer, from one complex in yeast to four in mammals. However, the physiological relevance of gene duplications for the vertebrate membrane-bound retromer subcomplex is unclear.

### Retromer Associates with the Dynactin Component p150^glued^

SNX5 and SNX6 have previously been shown to associate with a number of proteins including DOCK180, Mind bomb, Fanconi anemia complementation group A protein, and GIT1 (Cavet et al., 2008; Hara et al., 2008; Otsuki et al., 1999; Yoo et al., 2006). In the current study, we have established that SNX5 and SNX6 bind p150^glued^, a subunit of the minus-end-directed microtubule motor protein complex dynein-dynactin (Schroer, 2004; Vaughan and Vallee, 1995). We have validated this interaction by utilizing a number of experimental approaches, showing how it is required for efficient retromer-mediated endosome-to-TGN sorting of the CI-MPR. The resulting data add to the growing body of evidence that, by coupling to molecular motors, SNX-BARs coordinate membrane deformation with long-range transport between donor and recipient compartments (Cullen, 2008; Traer et al., 2007). Whether in vivo SNX-BAR mediated membrane tubulation requires coupling to a motor or whether this occurs independently through the ability of SNX-BARs to form oligomeric complexes remains unclear. SNX1 can induce membrane tubulation in vitro in the absence of other protein components (Carlton et al., 2004). Interestingly, we observed that suppression of p150^glued^ resulted in the formation of extended and relatively static GFP-SNX1-decorated tubules. This would be consistent with the association of a motor being required for generating longitudinal tension that, alongside a fission or constriction factor such as dynamin or EHD family members, assists the fission process that releases tubular carriers from the donor membrane (Daumke et al., 2007; Gokool et al., 2007; Roux et al., 2006).

These data establish that the retromer SNX subcomplex, in addition to generating and defining a tubular subdomain of the early endosome and acting as a docking site for the cargo-selective VPS26-VPS29-VPS35 subcomplex, also recruits the dynein-dynactin motor complex, providing a mechanism for coupling of tubular-based sorting with force generation and long-range, minus-end-directed microtubule-based transport.

### Association with Rab6IP1—a Mechanism by which Retromer-Labeled Transport Carriers Recognize Their Recipient Membrane

Important elements in membrane identity and recognition are the Rab GTPases (Behnia and Munro, 2005). Rab6 is a TGN-resident protein that, besides having roles in intra-Golgi and Golgi-to-ER transport (Girod et al., 1999; Martinez et al., 1997; White et al., 1999), is important in endosome-to-TGN retrograde transport of ricin and Shiga toxins, the latter being a known retromer cargo (Bujny et al., 2007; Del Nery et al., 2006; Monier et al., 2002; Popoff et al., 2007; Utskarpen et al., 2007). Binding partners of Rab6 include p150^glued^ (Short et al., 2002) and Rab6IP1 (Janoueix-Lerosey et al., 1995; Monier et al., 2002). The latter is a cytosolic factor that is associated with the Golgi through binding to Rab6, but can also associate with the endocytic recycling compartment marker Rab11 (Miserey-Lenkei et al., 2007). The only function in membrane trafficking ascribed to Rab6IP1 has been its proposed involvement in the tethering/docking of transport intermediates arriving at the TGN from endosomes (Miserey-Lenkei et al., 2007). It is worth noting that the human genome contains a close relative of Rab6IP1 termed DENN/MADD domain containing protein 5B (DENND5B) (these proteins are 70% identical and contain the same modular domain architecture). Nothing is known about the cell biology of this protein and hence the question of functional redundancy with Rab6IP1 remains an open one. However, the siRNAs used in the present study are predicted not to target DENND5B.

It has generally been thought that in a transport pathway, the recognition of the recipient membrane by tethering factors occurs after the isolated transport vesicle has uncoated (Behnia and Munro, 2005). In other words, the coat complex required for membrane deformation and cargo selection at the donor membrane does not play a role in recognition of the recipient membrane. The only exception to this has been the recent finding that the Sec23 component of the endoplasmic reticulum (ER) COPII coat complex associates with the TRAPPI tethering complex, an interaction that marks an ER-derived coated vesicle for fusion with the Golgi apparatus (Behnia et al., 2007; Cai et al., 2007). Our characterization of the association of SNX1 with the TGN-localized Rab6IP1, and its functional importance in retromer-mediated endosome-to-TGN transport, has identified another mechanism through which the destination of a transport vesicle can, in part, be dependent upon its associated coat complex. The SNX-BAR retromer coat complex therefore coordinates cargo sorting and tubular-based carrier formation at the donor membrane, with long-range transport and carrier recognition at the recipient membrane.

### Spatial Organization of the Retromer Endosomal Network and Its Importance For Sorting

Retromer-labeled early endosomes display a spatial organization defined by the presence of dispersed peripheral endosomes and an enrichment of endosomes in the vicinity of the TGN. From live cell imaging, it is readily apparent that this steady-state is highly dynamic. Retromer decorated vesicular and tubular structures undergo a number of fission and fusion events en route from the cell periphery to TGN proximal retromer-labeled vesicles. The steady-state distribution relies upon the interactions of retromer with p150^glued^ and Rab6IP1; suppression of either protein results in the generation of a new steady-state characterized by the peripheral dispersal of retromer vesicles and a correlated impairment in retromer-mediated sorting.

Nearly all hypotheses on endosomal sorting are based on positive discrimination, whereby the cytoplasmic tails of transmembrane cargos are selected by specific coat complexes and withdrawn from the bulk flow of cargo by sequestering into a bud that ultimately forms the isolated transport carrier. As we currently understand retromer function, the cargo-selective subcomplex concentrates cargo into the SNX-BAR defined membrane tubule prior to a fission event that generates the endosome-to-TGN carrier (Mari et al., 2008). The observed multiple fission and fusion events during the movement of peripheral retromer-labeled endosomes toward the TGN may therefore be viewed as corresponding to multiple retromer-mediated sorting events. Based upon such an interpretation (Figure S8), we propose that retromer-mediated sorting relies on iterative rounds of fission and fusion that progressively enrich retromer-labeled carriers with specific cargo as they migrate from the cell periphery toward TGN-juxtaposed endosomes. According to this model, it is from these TGN-proximal retromer-labeled endosomes that the actual process of endosome-to-TGN transport occurs. Altering the steady-state distribution of retromer-labeled endosomes therefore decreases the efficiency and quality of sorting, by perturbing the frequency and cargo-enrichment of retromer-coated vesicles that arrive at the TGN.

Overall, we have defined mechanistic details by which the SNX-BAR retromer coat complex coordinates cargo sorting and tubular-based carrier formation at the donor endosomal membrane with long-range transport and carrier recognition at the recipient TGN membrane.

## Experimental Procedures

### Antibodies

Mouse monoclonal antibodies directed against SNX1 (clone 51), SNX2 (clone 13), and p150^glued^ (clone1) were from BD Biosciences, Oxford, UK. Antibodies against SNX4 (C17), SNX5 (C15), SNX5 (D18), SNX6 (K18), SNX6 (N19), sulfotransferase (N20), CD63 (MEM259), and EEA1 (N19) were from Santa Cruz Biotech., Santa Cruz, CA. Mouse monoclonal, FITC conjugated CD8 antibody (clone 733) and the CI-MPR antibody (clone 2g11) were from Abcam, Cambridge, UK. Mouse monoclonal CD8 antibody (clone UCHT4) was from Ancell, Bayport, MN. Sheep polyclonal TGN46 and CIMPR monoclonal (clone MEM238) antibodies were from ABD, Kidlington, UK. Mouse monoclonal antibodies directed against alpha-Tubulin (clone DM1A), Flag epitope (clone M2), and GST (clone GST-2) and Flag-agarose gel (clone M2) were purchased from Sigma-Aldrich, Poole, UK. Rabbit polyclonal GFP antibody was a kind gift from Dr. Roland Kissmehl, University of Konstanz, Germany. Mouse monoclonal GFP antibody (mix of clones 7.1 and 13.1) was from Roche, Burgess Hill, UK. Rabbit polyclonal Giantin antibody was from Covance, Denver, PA.

### Yeast Two-Hybrid Screens

These were performed on a reamplified, Matchmaker III, pretransformed, adult human brain library as previously described (Traer et al., 2007).

### Immunoprecipitation

Culture dishes (15 cm) with HeLa or HEK293T cells at approximately 95% confluency were washed twice with ice-cold PBS and either lysed hypotonically by incubation with 20 mM HEPES (pH 7.2) followed by scraping, dounce homogenization, and supplementation with salt to a final salt concentration of 50 mM KCl for the IP of p150^glued^ or by the addition of lysis buffer containing 20 mM HEPES (pH 7.2), 100 mM K-acetate, 2.5 mM Mg-acetate, 0.1% Triton X-100, and scraping for all other IPs. Lysates were cleared by centrifugation at 16,000 × g for 20 min at 4°C. Antibody (1 μg) was added to the cleared lysate per condition, incubated for 1 hr at 4°C on a roller mixer SRT2 (Stuart Scientific, Stone, UK), and followed by incubation with G-agarose (Upstate, Chandlers Ford, UK) equilibrated in lysis buffer. Precipitates were washed once in lysis buffer and three to four times in detergent-free lysis buffer. Complexes were eluted from the G-agarose by boiling in NuPAGE LDS sample buffer (Invitrogen, Paisley, UK) and subjected to gel electrophoresis and western blotting.

### Production of Lentiviruses

These were carried out as described (Danson et al., 2007; Demaison et al., 2002). Plasmids for lentiviral production were kind gifts of Dr. Giles Cory, University of Bristol, UK.

### Transient Transfection of HEK293T Cells Using Ethylene Imine Polymer

This was performed as previously described (Danson et al., 2007). Briefly, a total of 90 μg plasmid DNA was transfected per 15 cm dish HEK293T cells at approximately 95% confluency. The following day cells were subjected to IP or GST pull-down.

### GST Pull-Downs

These were essentially as described (Traer et al., 2007), with a modification used for Figure 2B in which GST-fusion proteins were expressed in HEK293T cells in order to probe for coisolation of endogenous p150^glued^, other dynein/dynactin subunits, and the retromer subunits SNX1 and SNX2. For Rab6IP1 we used the method described by (Monier et al., 2002).

### Live- and Fixed-Cell Imaging

This utilized the TCS-SP2 and TCS-SP5 confocal imaging systems (both Leica, Heidelberg, Germany) and UltraView MultiUser Confocal Optical Scanner (Perkin-Elmer, Waltham, MA), essentially as described (Wassmer et al., 2007). For tracking of vesicles, we used the ImageJ plug-in “Manual Tracking.”

### Transient Transfection of HeLa Cells with siRNAs

The relevant siRNAs (Dharmacon, Lafayette, CO) were transiently transfected using HiPerFect (QIAGEN, Hilden, Germany) according to the manufacturer's instruction. Final concentration of siRNA duplexes was 20 nM. Cells were analyzed for phenotypes 48–72 hr after transfection. siRNAs can be found in Table S1.

### Steady-State Distribution of CI-MPR

Ten fields of vision per siRNA treatment, carried out in triplicate, were imaged under identical conditions using a Leica TCS-SP2 confocal imaging system at 400× magnification in the CI-MPR and the giantin channels, with the latter defining Golgi positions, numbers, and sizes. ImageJ was used for data analysis. Using maximum Z projections, a half maximal cut off was defined for the CI-MPR stain, and the number of cells above this threshold were counted using the “analyze particles” command and calculated as a percentage of total cells.

### Kinetics of CD8 Uptake

This was performed as described previously (Wassmer et al., 2007). Images, taken using 400× magnification using a Leica TCS-SP2, were analyzed using ImageJ. The percentage of the CD8-channel pixel intensities in the TGN area as defined by TGN46 staining was calculated on a maximum Z projection per field of vision. Ten fields of vision per siRNA were imaged for the time points 30 and 45 min, 5 fields of vision per siRNA for time point 15 min, and 3 fields of vision for time point 0 min.

### Quantification of Endogenous Steady-State Distribution of SNX1

This was performed as the CD8 uptake analysis, with SNX1 and TGN46 in place of CI-MPR and giantin, respectively, and with the calculated ratio TGN-proximal/non-TGN-proximal.

### Electron Microscopy

Cryoimmunoelectron microscopy was performed as described (Carlton et al., 2004; Mari et al., 2008). HeLa cells expressing GFP-Rab6IP1 were fixed in 4% paraformaldehyde/0.05% glutaraldehyde in 0.1 M phosphate buffer. The fixed cells were scraped off the dish in 1% gelatine in phosphate buffer and spun down in 10% gelatine. After 1 hr of solidification of the gelatine on ice, the pellets were cut into small blocks and infiltrated with 2.3 M sucrose at 4°C overnight. The blocks were mounted on aluminum pins and frozen in liquid nitrogen for ultrathin cryosectioning. Sections (70 nm) were collected at −120°C in 1% methyl-cellulose in 1.2 M sucrose on formvar/carbon-coated copper mesh grids. The sections were labeled with monoclonal mouse anti-SNX1 and polyclonal rabbit anti-GFP (Molecular Probes Invitrogen, Carlsbad, CA) antibodies and secondary antibodies anti-mouse 6 nm gold and anti-rabbit 10 nm gold (Aurion, Wageningen, The Netherlands). The sections were counterstained with 0.3% uranyl acetate in 1.8% methyl-cellulose and imaged on a FEI Tecnai 12 Biotwin transmission electron microscope equipped with a bottom mount.

### *C. elegans* Genetics

General methods for culture, manipulation, and genetics of *C. elegans* were as described (Lewis and Fleming, 1995). Strains were cultured at 20°C. Embryos were isolated using sodium hypochlorite treatment and were grown for 2 days at 25°C. The final positions of the left Q descendants was scored using a *mec-7::gfp* (*muIs32*) reporter transgene (Ch'ng et al., 2003). The *dnc-1(or404ts)* allele was provided by the *Caenorhabditis* Genetics Center (University of Minnesota, Minneapolis, MN).

## Supplemental Data

Document S1. Two Tables and Eight Figures

Movie S1. Live Cell Imaging of GFP-p150glued (Green) and mCherry-SNX6 (Red), a Time Course of which Is Presented in Figure 2C Both transgenes were expressed in HeLa cells using lentiviruses at low levels. Please note the high degree of co-localisation and co-movement of tubular and vesicular structures labelled with both p150^glued^ and SNX6. An image was taken each 2.67 seconds and the video converted to display 5 frames a second, resulting in ∼13 x real time. For scale bar compare Figure 2C.

Movie S2. Visualisation of Transport of CD8-Tagged CI-MPR to the TGN HeLaM cells expressing both CD8-CI-MPR and mCherry-Rab6a′ were labelled by adding a FITC-tagged CD8 antibody into the culture medium, immediately followed by confocal live cell imaging. In Movie S2A the FITC channel is displayed, in Movie S2B the corresponding mCherry channel and in Movie S2C the merge of both channels (FITC – green, mCherry – red). Please note the accumulation of CD8-FITC label in the mCherry-Rab6a′ decorated Golgi area over the 20 min time period imaged. Time and scale bar as indicated in the video.

Movie S3. Retromer Decorated Vesicles Stained by GFP-SNX6 Undergo Sporadic Movement towards the Vicinity of mCherry-Rab6a′ Labeled Golgi Movie S3A. GFP-SNX6. Movie S3B. mCherry-Rab6a′. Movie S3C. Merge of the two channels, GFP – green, mCherry – red. The video was collected over a 5 min time period and is played at ∼17 x real time. Numerous events are visible in which SNX6 labelled vesicles move in all directions, but with a perceived tendency to travel towards the Golgi, where an accumulation of bright SNX6 stained vesicles can be observed. For scale bar and quantification, compare Figure 4A and C.

Movie S4. Retromer Labeled Vesicles and Tubules Display a Complex Pattern of Movement Involving Numerous Events of Label Merging and Splitting GFP-SNX6 was lentivirally transfected into HeLa cells and imaged over a 5 min time period (video at ∼17 x real time). The most likely explanation of label merging is fusion of vesicles, while label splitting could be interpreted as fission event. See Figure 4B for scale bar.

Movie S5. Suppression of p150glued Reduces Efficient Movement towards the TGN Movie S5A. GFP-SNX6. Movie S5B. mCherry-Rab6a′. Movie S5C. Merge of the two channels, GFP – green, mCherry – red. Recording time of the video was 5 min, played at ∼17 x real time. While chaotic movement of vesicles is still abundant under p150^glued^ suppression, their trajectories are no longer directed towards the TGN. By consequence, the concentration of retromer positive vesicles at the TGN is virtually abolished. Compare Figure 4A and C for scale bar and quantification.

Movie S6. Rab6IP1 Is Localized at the TGN with Close Connection to Numerous CD8-CI-MPR Positive Carriers CD8-FITC labelled antibody (green) present in the culture medium trafficked to the mCherry-Rab6IP1 (red) stained TGN. Images were collected at one image per 2.1 s (movie: ∼10 x real time). Please note the mCherry-Rab6IP1 decorated fibre that emerges from the TGN to which a CD8-FITC positive carrier seems to be attached, as the swaying of the fibre is parallelled by the movement of the carrier. Unfortunately, the red channel bleaches rapidly, as a confocal scanning microscope was used. For scale bar see Figure 6A.

## References

[bib1] Arighi C.N., Hartnell L.M., Aguilar R.C., Haft C.R., Bonifacino J.S. (2004). Role of the mammalian retromer in sorting of the cation-independent mannose 6-phosphate receptor. J. Cell Biol..

[bib2] Banziger C., Soldini D., Schutt C., Zipperlen P., Hausmann G., Basler K. (2006). Wntless, a conserved membrane protein dedicated to the secretion of Wnt proteins from signaling cells. Cell.

[bib3] Bartscherer K., Pelte N., Ingelfinger D., Boutros M. (2006). Secretion of Wnt ligands requires Evi, a conserved transmembrane protein. Cell.

[bib4] Behnia R., Munro S. (2005). Organelle identity and the signposts for membrane traffic. Nature.

[bib5] Behnia R., Barr F.A., Flanagan J.J., Barlowe C., Munro S. (2007). The yeast orthologue of GRASP65 forms a complex with a coiled-coil protein that contributes to ER to Golgi traffic. J. Cell Biol..

[bib6] Bonifacino J.S., Hurley J.H. (2008). Retromer. Curr. Opin. Cell Biol..

[bib7] Bujny M.V., Popoff V., Johannes L., Cullen P.J. (2007). The retromer component sorting nexin-1 is required for efficient retrograde transport of Shiga toxin from early endosome to the trans Golgi network. J. Cell Sci..

[bib8] Cai H., Yu S., Menon S., Cai Y., Lazarova D., Fu C., Reinisch K., Hay J.C., Ferro-Novick S. (2007). TRAPPI tethers COPII vesicles by binding the coat subunit Sec23. Nature.

[bib9] Carlton J., Bujny M., Peter B.J., Oorschot V.M., Rutherford A., Mellor H., Klumperman J., Mcmahon H.T., Cullen P.J. (2004). Sorting nexin-1 mediates tubular endosome-to-TGN transport through coincidence sensing of high- curvature membranes and 3-phosphoinositides. Curr. Biol..

[bib10] Carlton J.G., Bujny M.V., Peter B.J., Oorschot V.M., Rutherford A., Arkell R.S., Klumperman J., Mcmahon H.T., Cullen P.J. (2005). Sorting nexin-2 is associated with tubular elements of the early endosome, but is not essential for retromer-mediated endosome-to-TGN transport. J. Cell Sci..

[bib11] Cavet M.E., Pang J., Yin G., Berk B.C. (2008). An epidermal growth factor (EGF) -dependent interaction between GIT1 and sorting nexin 6 promotes degradation of the EGF receptor. FASEB J..

[bib12] Ch'ng Q., Williams L., Lie Y.S., Sym M., Whangbo J., Kenyon C. (2003). Identification of genes that regulate a left-right asymmetric neuronal migration in Caenorhabditis elegans. Genetics.

[bib13] Coudreuse D.Y., Roel G., Betist M.C., Destree O., Korswagen H.C. (2006). Wnt gradient formation requires retromer function in Wnt-producing cells. Science.

[bib14] Cullen P.J. (2008). Endosomal sorting and signalling: an emerging role for sorting nexins. Nat. Rev. Mol. Cell Biol..

[bib15] Danson C.M., Pocha S.M., Bloomberg G.B., Cory G.O. (2007). Phosphorylation of WAVE2 by MAP kinases regulates persistent cell migration and polarity. J. Cell Sci..

[bib16] Daumke O., Lundmark R., Vallis Y., Martens S., Butler P.J., McMahon H.T. (2007). Architectural and mechanistic insights into an EHD ATPase involved in membrane remodelling. Nature.

[bib17] Del Nery E., Miserey-Lenkei S., Falguieres T., Nizak C., Johannes L., Perez F., Goud B. (2006). Rab6A and Rab6A′ GTPases play non-overlapping roles in membrane trafficking. Traffic.

[bib18] Demaison C., Parsley K., Brouns G., Scherr M., Battmer K., Kinnon C., Grez M., Thrasher A.J. (2002). High-level transduction and gene expression in hematopoietic repopulating cells using a human immunodeficiency [correction of imunodeficiency] virus type 1-based lentiviral vector containing an internal spleen focus forming virus promoter. Hum. Gene Ther..

[bib19] Derby M.C., Lieu Z.Z., Brown D., Stow J.L., Goud B., Gleeson P.A. (2007). The trans-Golgi network golgin, GCC185, is required for endosome-to-Golgi transport and maintenance of Golgi structure. Traffic.

[bib20] Driskell O.J., Mironov A., Allan V.J., Woodman P.G. (2007). Dynein is required for receptor sorting and the morphogenesis of early endosomes. Nat. Cell Biol..

[bib21] Encalada S.E., Willis J., Lyczak R., Bowerman B. (2005). A spindle checkpoint functions during mitosis in the early *Caenorhabditis elegans* embryo. Mol. Biol. Cell.

[bib22] Franch-Marro X., Wendler F., Guidato S., Griffith J., Baena-Lopez A., Itasaki N., Maurice M.M., Vincent J.P. (2008). Wingless secretion requires endosome-to-Golgi retrieval of Wntless/Evi/Sprinter by the retromer complex. Nat. Cell Biol..

[bib23] Frost A., Perera R., Roux A., Spasov K., Destaing O., Egelman E.H., De Camilli P., Unger V.M. (2008). Structural basis of membrane invagination by F-BAR domains. Cell.

[bib24] Girod A., Storrie B., Simpson J.C., Johannes L., Goud B., Roberts L.M., Lord J.M., Nilsson T., Pepperkok R. (1999). Evidence for a COP-I-independent transport route from the Golgi complex to the endoplasmic reticulum. Nat. Cell Biol..

[bib25] Gokool S., Tattersall D., Seaman M.N. (2007). EHD1 interacts with retromer to stabilize SNX1 tubules and facilitate endosome-to-Golgi retrieval. Traffic.

[bib26] Griffin C.T., Trejo J., Magnuson T. (2005). Genetic evidence for a mammalian retromer complex containing sorting nexins 1 and 2. Proc. Natl. Acad. Sci. USA.

[bib27] Gullapalli A., Garrett T.A., Paing M.M., Griffin C.T., Yang Y., Trejo J. (2004). A role for sorting nexin 2 in epidermal growth factor receptor down-regulation: evidence for distinct functions of sorting nexin 1 and 2 in protein trafficking. Mol. Biol. Cell.

[bib28] Hara S., Kiyokawa E., Iemura S., Natsume T., Wassmer T., Cullen P.J., Hiai H., Matsuda M. (2008). The DHR1 domain of DOCK180 binds to SNX5 and regulates cation-independent mannose 6-phosphate receptor transport. Mol. Biol. Cell.

[bib29] Horazdovsky B.F., Davies B.A., Seaman M.N., Mclaughlin S.A., Yoon S., Emr S.D. (1997). A sorting nexin-1 homologue, Vps5p, forms a complex with Vps17p and is required for recycling the vacuolar protein-sorting receptor. Mol. Biol. Cell.

[bib30] Janoueix-Lerosey I., Jollivet F., Camonis J., Marche P.N., Goud B. (1995). Two-hybrid system screen with the small GTP-binding protein Rab6. Identification of a novel mouse GDP dissociation inhibitor isoform and two other potential partners of Rab6. J. Biol. Chem..

[bib31] Johansson M., Rocha N., Zwart W., Jordens I., Janssen L., Kuijl C., Olkkonen V.M., Neefjes J. (2007). Activation of endosomal dynein motors by stepwise assembly of Rab7-RILP-p150Glued, ORP1L, and the receptor betalll spectrin. J. Cell Biol..

[bib32] Jordens I., Fernandez-Borja M., Marsman M., Dusseljee S., Janssen L., Calafat J., Janssen H., Wubbolts R., Neefjes J. (2001). The Rab7 effector protein RILP controls lysosomal transport by inducing the recruitment of dynein-dynactin motors. Curr. Biol..

[bib33] Lewis J.A., Fleming J.T. (1995). Basic culture methods. Methods Cell Biol..

[bib34] Mari M., Bujny M.V., Zeuschner D., Geerts W.J., Griffith J., Petersen C.M., Cullen P.J., Klumperman J., Geuze H.J. (2008). SNX1 defines an early endosomal recycling exit for sortilin and mannose 6-phosphate receptors. Traffic.

[bib35] Martinez O., Antony C., Pehau-Arnaudet G., Berger E.G., Salamero J., Goud B. (1997). GTP-bound forms of rab6 induce the redistribution of Golgi proteins into the endoplasmic reticulum. Proc. Natl. Acad. Sci. USA.

[bib36] McMahon H.T., Gallop J.L. (2005). Membrane curvature and mechanisms of dynamic cell membrane remodelling. Nature.

[bib37] Miserey-Lenkei S., Waharte F., Boulet A., Cuif M.H., Tenza D., El Marjou A., Raposo G., Salamero J., Heliot L., Goud B., Monier S. (2007). Rab6-interacting protein 1 links Rab6 and Rab11 function. Traffic.

[bib38] Monier S., Jollivet F., Janoueix-Lerosey I., Johannes L., Goud B. (2002). Characterization of novel Rab6-interacting proteins involved in endosome-to-TGN transport. Traffic.

[bib39] Otsuki T., Kajigaya S., Ozawa K., Liu J.M. (1999). SNX5, a new member of the sorting nexin family, binds to the Fanconi anemia complementation group A protein. Biochem. Biophys. Res. Commun..

[bib40] Pan C.L., Baum P.D., Gu M., Jorgensen E.M., Clark S.G., Garriga G. (2008). C. elegans AP-2 and retromer control Wnt signaling by regulating mig-14/Wntless. Dev. Cell.

[bib41] Popoff V., Mardones G.A., Tenza D., Rojas R., Lamaze C., Bonifacino J.S., Raposo G., Johannes L. (2007). The retromer complex and clathrin define an early endosomal retrograde exit site. J. Cell Sci..

[bib42] Port F., Kuster M., Herr P., Furger E., Banziger C., Hausmann G., Basler K. (2008). Wingless secretion promotes and requires retromer-dependent cycling of Wntless. Nat. Cell Biol..

[bib43] Rojas R., Kametaka S., Haft C.R., Bonifacino J.S. (2007). Interchangeable but essential functions of SNX1 and SNX2 in the association of retromer with endosomes and the trafficking of mannose 6-phosphate receptors. Mol. Cell. Biol..

[bib44] Roux A., Uyhazi K., Frost A., De Camilli P. (2006). GTP-dependent twisting of dynamin implicates constriction and tension in membrane fission. Nature.

[bib45] Schroer T.A. (2004). Dynactin. Annu. Rev. Cell Dev. Biol..

[bib46] Seaman M.N. (2004). Cargo-selective endosomal sorting for retrieval to the Golgi requires retromer. J. Cell Biol..

[bib47] Seaman M.N. (2005). Recycling your receptors with retromer. Trends Cell Biol..

[bib48] Seaman M.N. (2007). Identification of a novel conserved sorting motif required for retromer-mediated endosome-to-TGN retrieval. J. Cell Sci..

[bib49] Seaman M.N., McCaffery J.M., Emr S.D. (1998). A membrane coat complex essential for endosome-to-Golgi retrograde transport in yeast. J. Cell Biol..

[bib50] Seet L.F., Hong W. (2006). The Phox (PX) domain proteins and membrane traffic. Biochim. Biophys. Acta.

[bib51] Short B., Preisinger C., Schaletzky J., Kopajtich R., Barr F.A. (2002). The Rab6 GTPase regulates recruitment of the dynactin complex to Golgi membranes. Curr. Biol..

[bib52] Teasdale R.D., Loci D., Houghton F., Karlsson L., Gleeson P.A. (2001). A large family of endosome-localized proteins related to sorting nexin 1. Biochem. J..

[bib53] Traer C.J., Rutherford A.C., Palmer K.J., Wassmer T., Oakley J., Attar N., Carlton J.G., Kremerskothen J., Stephens D.J., Cullen P.J. (2007). SNX4 coordinates endosomal sorting of TfnR with dynein-mediated transport into the endocytic recycling compartment. Nat. Cell Biol..

[bib54] Utskarpen A., Slagsvold H.H., Dyve A.B., Skanland S.S., Sandvig K. (2007). SNX1 and SNX2 mediate retrograde transport of Shiga toxin. Biochem. Biophys. Res. Commun..

[bib55] Vaughan K.T., Vallee R.B. (1995). Cytoplasmic dynein binds dynactin through a direct interaction between the intermediate chains and p150Glued. J. Cell Biol..

[bib56] Wassmer T., Attar N., Bujny M.V., Oakley J., Traer C.J., Cullen P.J. (2007). A loss-of-function screen reveals SNX5 and SNX6 as potential components of the mammalian retromer. J. Cell Sci..

[bib57] Whangbo J., Kenyon C. (1999). A Wnt signaling system that specifies two patterns of cell migration in C. elegans. Mol. Cell.

[bib58] White J., Johannes L., Mallard F., Girod A., Grill S., Reinsch S., Keller P., Tzschaschel B., Echard A., Goud B., Stelzer E.H. (1999). Rab6 coordinates a novel Golgi to ER retrograde transport pathway in live cells. J. Cell Biol..

[bib59] Yang P.T., Lorenowicz M.J., Silhankova M., Coudreuse D.Y., Betist M.C., Korswagen H.C. (2008). Wnt signaling requires retromer-dependent recycling of MIG-14/Wntless in Wnt-producing cells. Dev. Cell.

[bib60] Yoo K.W., Kim E.H., Jung S.H., Rhee M., Koo B.K., Yoon K.J., Kong Y.Y., Kim C.H. (2006). Snx5, as a Mind bomb-binding protein, is expressed in hematopoietic and endothelial precursor cells in zebrafish. FEBS Lett..

[bib61] Zhang H., Skop A.R., White J.G. (2008). Src and Wnt signaling regulate dynactin accumulation to the P2-EMS cell border in *C. elegans* embryos. J. Cell Sci..

