# Supplementary material for: The Retromer Coat Complex Coordinates Endosomal Sorting and Dynein-Mediated Transport, with Carrier Recognition by the trans-Golgi Network
Source: Dev Cell. 2009 Jul 21;17(1):110–22. doi: 10.1016/j.devcel.2009.04.016 (PMC2714578; doi:10.1016/j.devcel.2009.04.016)
Supplement: Document S1. Two Tables and Eight Figures [file mmc1.pdf]

Supplemental Data

The Retromer Coat Complex Coordinates Endosomal Sorting and Dynein-Mediated Transport, with Carrier Recognition by the *trans*-Golgi Network

Thomas Wassmer, Naomi Attar, Martin Harterink, Jan R.T. van Weering, Colin J. Traer, Jacqueline Oakley, Bruno Goud, David J. Stephens, Paul Verkade, Hendrik C. Korswagen, and Peter J. Cullen

Table S1. All Clones Isolated as "Positive" Interactions from the Retromer SNX-BAR Yeast Two-Hybrid Analysis

Yeast two-hybrid bait	*Identified interactor*	*Number of hits in screen*	*Accession number*
**Sorting nexin-1**	Sorting nexin-6	6	AF121856
	ADP-ribosylation factor-like 6 interacting protein 1	6	NM_015161
	Sorting nexin-5	5	NM_152227
	Yip1 domain family, member 5, YPT-interacting protein 1 A	2	NM_030799
	RAB6 interacting protein 1	1	NM_015213
	neuronal pentraxin receptor	1	NM_014293
	LOC152217 protein	1	XR_017713
	zinc finger, ZZ-type containing 3, isoform CRA_e	1	NM_015534
	suppressor of hairy wing homolog 2	1	NM_080764
**Sorting nexin-2**	Sorting nexin-6	3	AF121856
	Zinc finger CDGSH	2	NP_060934.1
	Archain	2	NM_001655
	Sorting nexin-5	1	NM_152227
**Sorting nexin-5**	Sorting nexin-1	24	NM_003099
	Sorting nexin-2	6	NM_003100
	CREB/ATF bZIP transcription factor	5	BC060807
	p150glued	4	NM_004082
	Sorting nexin-6	4	AF121856
	Sorting nexin-5	1	NM_152227
	Stathmin-2 (SCG10 protein)	1	AAV38837
	CSE1 chromosome segregation 1-like protein	1	NM_001316
**Sorting nexin-6**	p150glued	16	NM_004082

	Sorting nexin-1	14	NM_003099
	Sorting nexin-2	6	NM_003100
	MLLT10	2	NM_004641
	Nudel	2	NM_001025579
	creatine kinase, brain, isoform CRA_b	1	NM_001823
	chromodomain helicase DNA binding protein 9, isoform CRA_b	1	CH471092
	microtubule-associated protein 1B isoform 2	1	NM_032010
	zinc finger, A20 domain containing 2, isoform CRA_c	1	EAW62535
	vacuolar H+ ATPase G1	1	NM_004888
	bromodomain adjacent to zinc finger domain, 2B, isoform CRA_c, homo sapiens	1	NM_013450
	nephrocystin 3	1	NM_153240
	protein tyrosine phosphatase, receptor type, J, isoform CRA_d	1	NM_002843
	CREB/ATF bZIP transcription factor	1	BC060807
	PREDICTED: similar to angiomotin	1	XM_941912
	C10orf78 protein [Homo sapiens]	1	NM_001002759
	ADP-ribosylation-like factor 6 interacting protein 5 variant	1	NM_006407

Table S2. Sirna Sequences Used during This Study

siRNA name	Target sequence
control	gacaagaaccagaacgcca
p150glued-I	ctggagcgctgtatcgtaa
p150glued-II	gaagatcgagagacagtta
p150glued-III	gctcatgcctcgtctcatt
Rab6IP1-I	gcacaaacctattatgaga
Rab6IP1-II	gcaaatcgtttcactcaga
Rab6IP1-III	gaacacctgcgtttagata
SNX1	gaacaagaccaagagccac

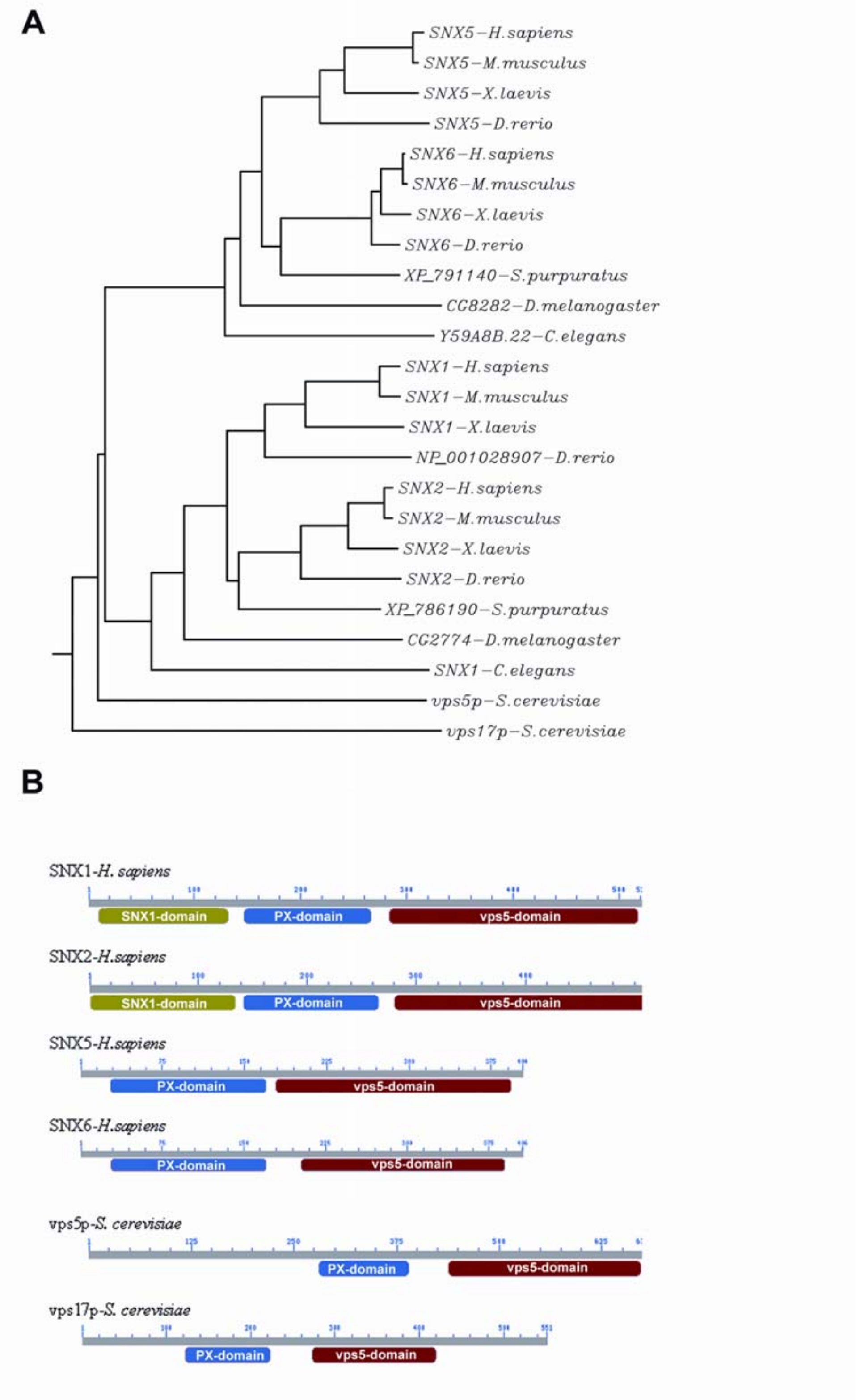

Figure S1. Phylogenetic Analysis of Retromer Snxs Suggests that Vertebrate SNX1/SNX2 and SNX5/SNX6 Arose by Duplication from an Invertebrate SNX Gene Pair

(A) Protein sequences of retromer SNXs of *Homo sapiens, Mus musculus, Xenopus laevis, Danio rerio, Strongylocentrotus purpuratus, Drosophila melanogaster, Caenorhabditis elegans* and *Saccharomyces cerevisiae* were either retrieved from databases or identified by blast searches. A Clustal W alignment was performed and a N-J tree constructed (http://align.genome.jp).

(B) Comparison of domain structure in the human and *S. cerevisiae* retromer SNXs.

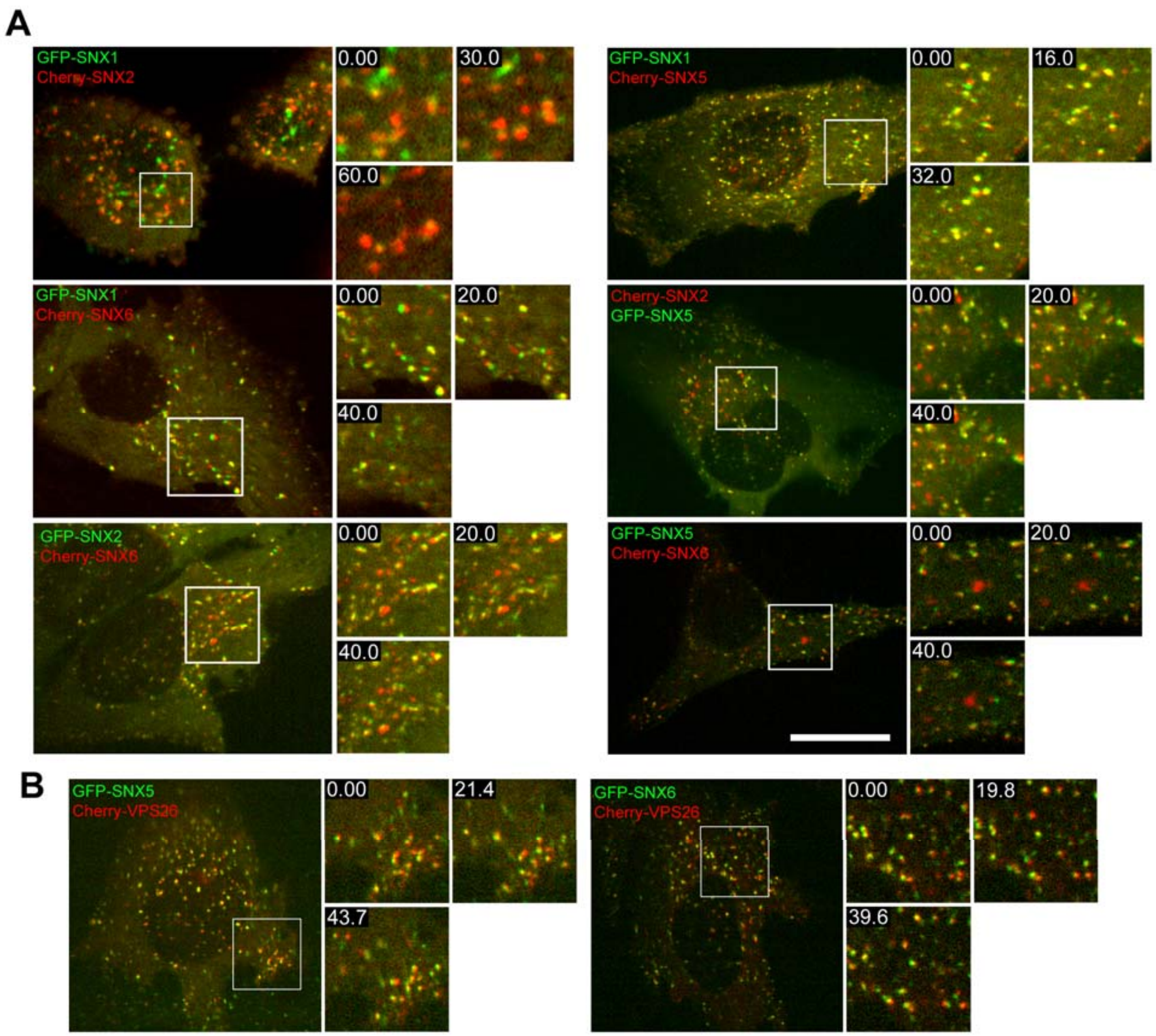

Figure S2. Co-Expression of GFP or mcherry-Tagged Retromer Subunits Reveals High Degrees of Co-Localisation and Co-Movement

(A) Co-localisation analysis was performed by co-expression of all possible pairings of the four retromer SNXs (SNX1, SNX2, SNX5 and SNX6) by lentiviral transduction of HeLa cells and live-cell imaging. In all SNX-combinations co-localisation and co-movement of vesicular structures can be detected. However, co-localisation is particularly pronounced for the combinations GFP-SNX1/mCherry-SNX5, GFP-SNX1/mCherry-SNX6, mCherry-SNX2/GFP-SNX5 and GFP-SNX2/mCherry-SNX6.

(B) Co-expression of a marker of the cargo-selective sub-complex of the retromer (mCherry-VPS26) with GFP-tagged SNX5 or SNX6 displays a high degree of co-localisation. Please note that the images in the GFP- and the mCherry-channel were collected sequentially, leading to the mCherry-channel being recorded with a short delay relative to the GFP-channel. Therefore the red and the green label on very fast moving structures appear to be separate, while most likely they reside on the same structure. Bar, 20 µm.

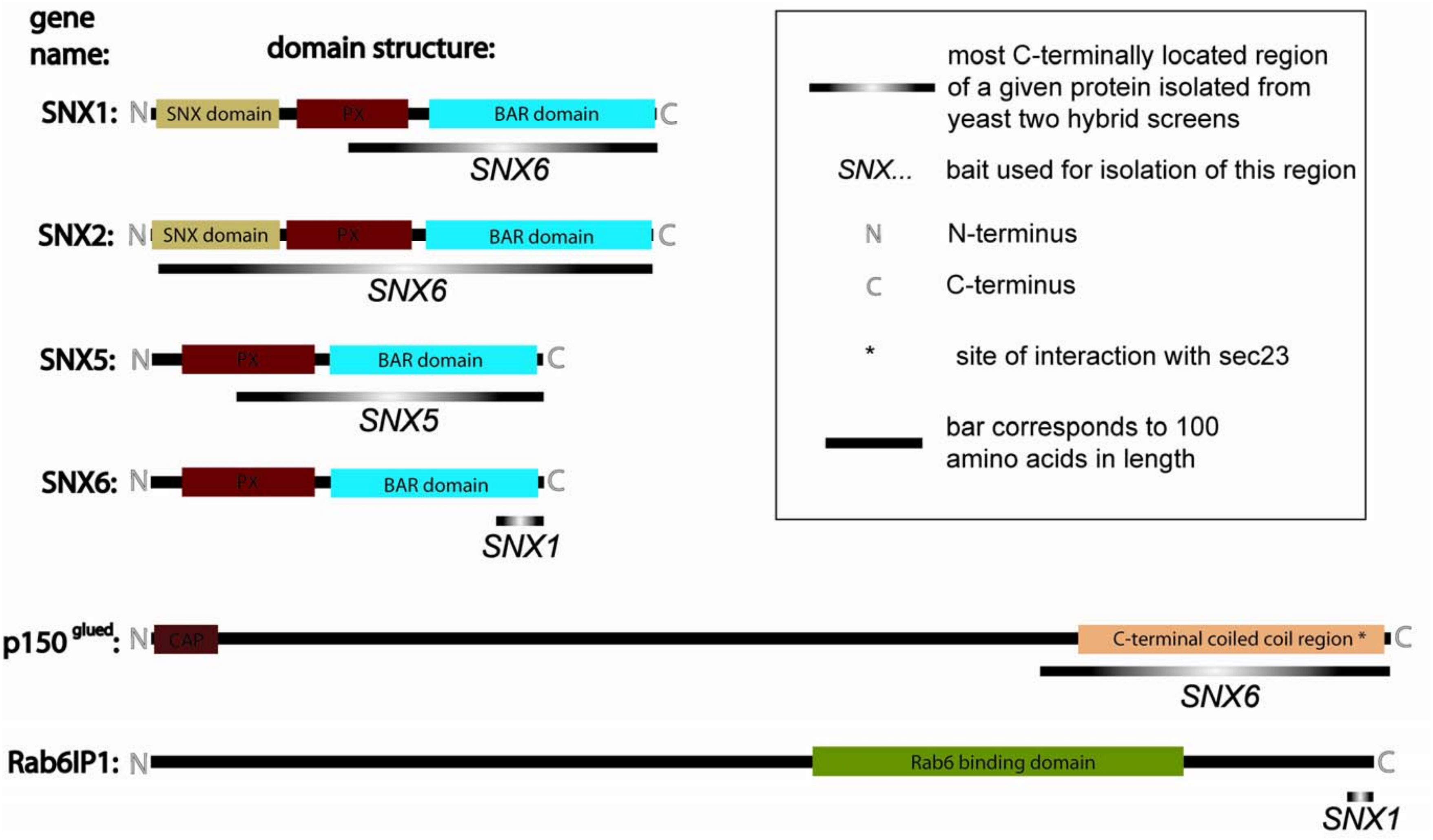

Figure S3. Schematic Representation of Yeast Two Hybrid Hits with Identified Interaction Regions

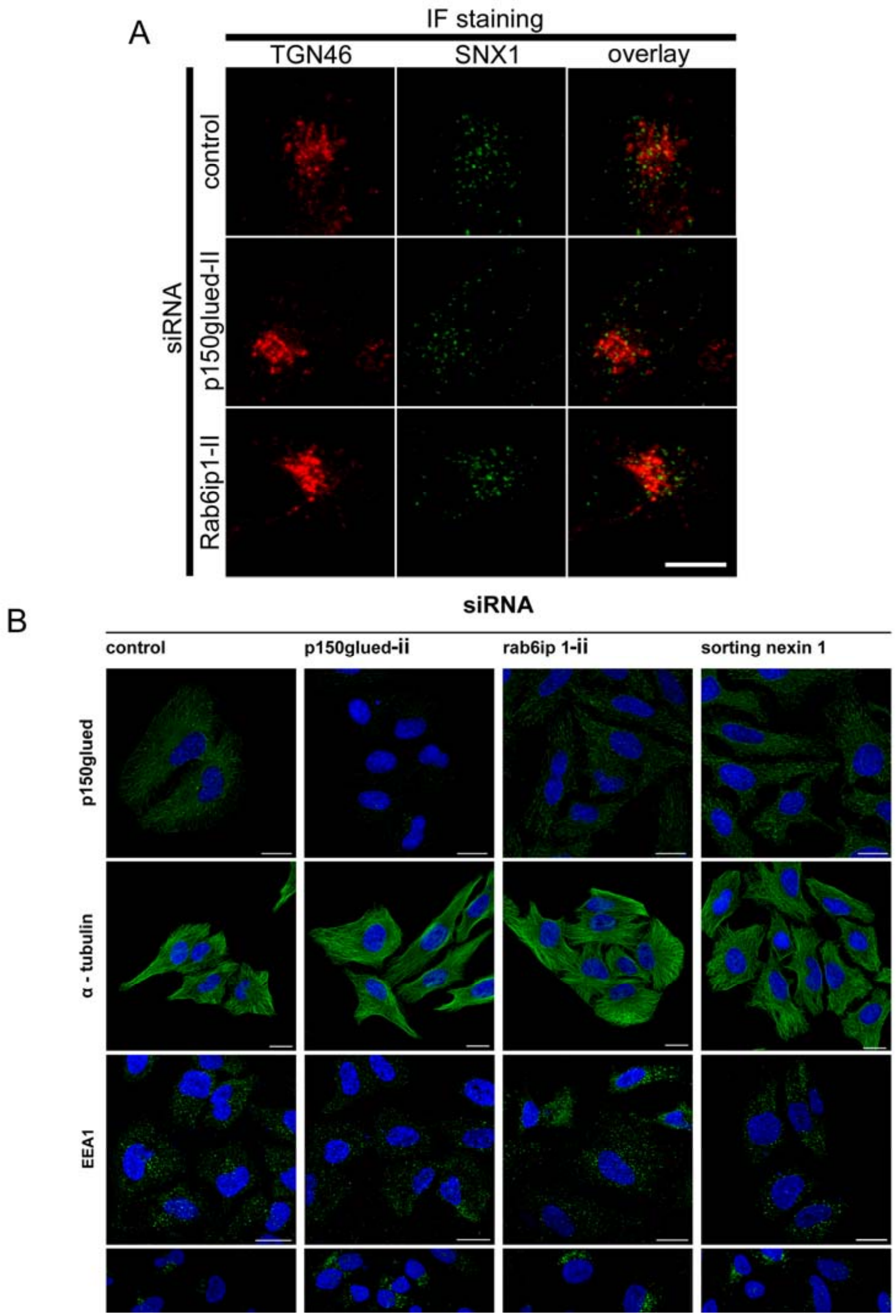
A
IF staining
TGN46
SNX1
overlay
siRNA
control
p150glued-II
Rab6ip1-II
B
siRNA
control
p150glued-ii
rab6ip 1-ii
sorting nexin 1
p150glued
α - tubulin
EEA1

Figure S4. Cellular Organization upon p150^{glued} and Rab6IP1 Suppression
Under the level of p150^{glued} or Rab6IP1 suppression achieved in the current study, the organisation of the TGN, as shown by labelling TGN46 is not grossly perturbed nor is the microtubule organisation or distribution of EEA1-positive early endosomes. A minor dispersal of the CD63-labelled lysosomal compartment is apparent upon p150^{glued} suppression. Bars, 20 µm.

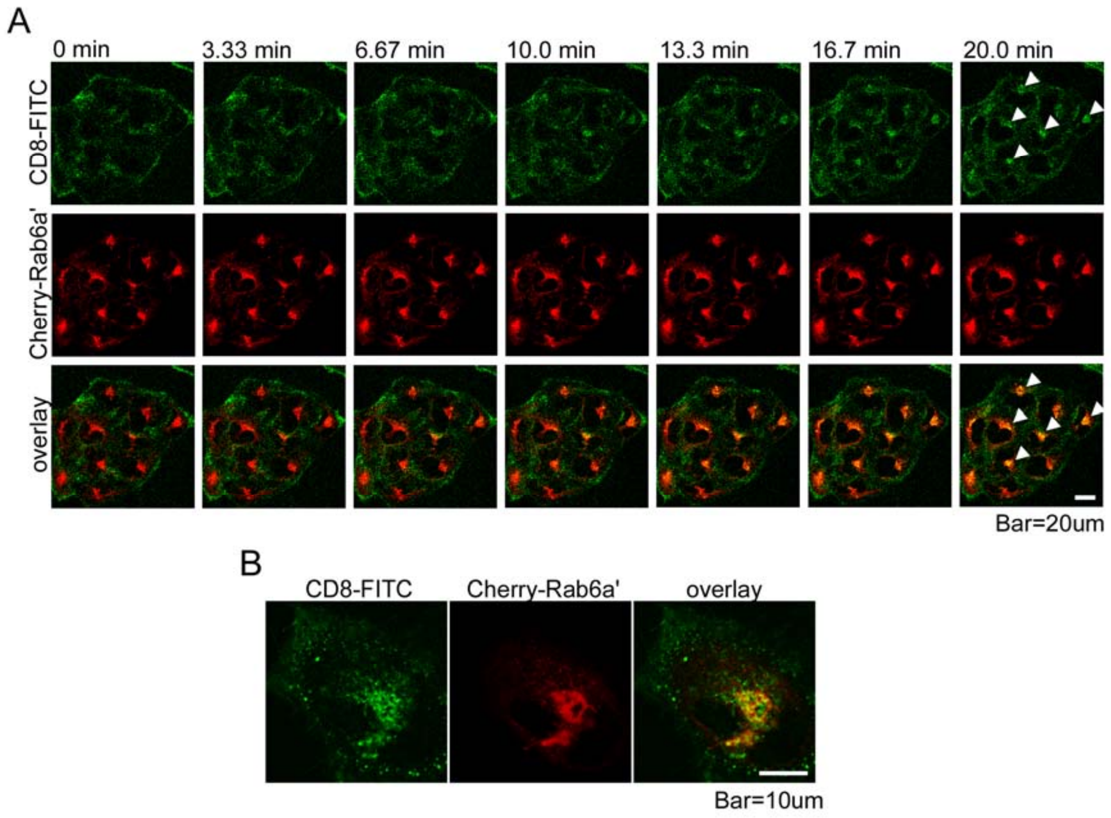

Figure S5. Analyzing the Kinetics of CD8-CI-MPR Transport
(A) CD8-tagged CI-MPR labelled with a CD8-FITC antibody travels from the plasma membrane via early endosomes to the TGN, as illustrated with stills taken from a 20 min movie. mCherry-Rab6a' labels the Golgi complex, arrowheads indicate CD8-FITC label concentration in the Golgi area (see Movie S2). Bar, 20 µm.
(B) Enlarged display of a HeLa cell after 20 min of CD8-FTIC uptake. Bar, 10µm.

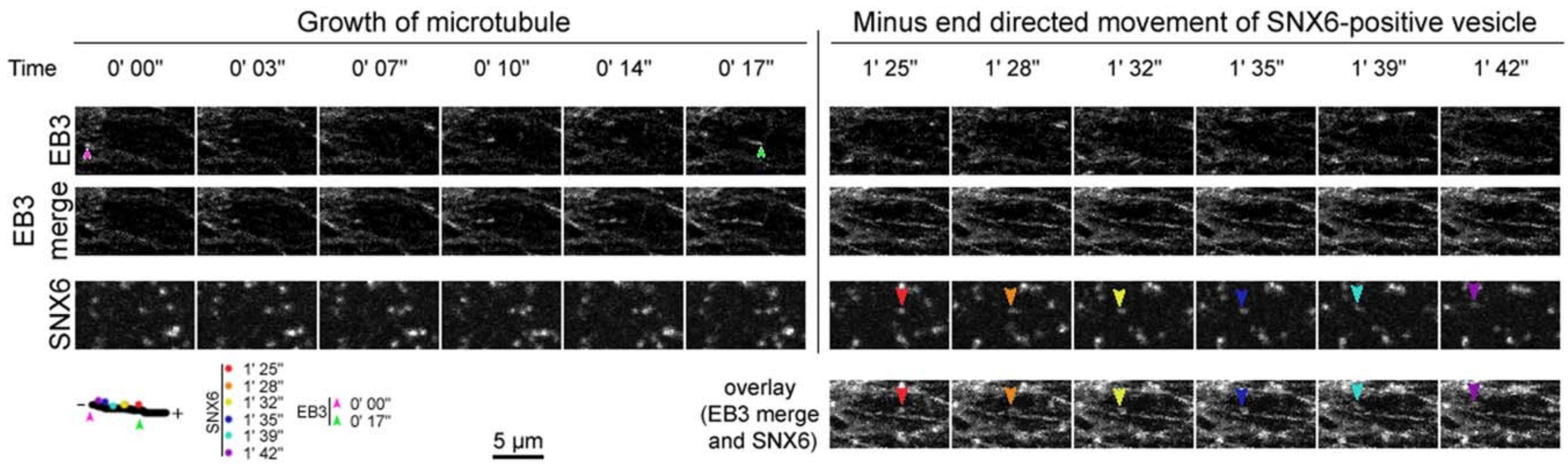

Figure S6. SNX6-Labelled Endosomes Are Observed to Undergo Microtubule Minus-End Directed Movement
Images captured from a movie examining the relationship between GFP-tagged EB3 (plus-end microtubule tracking protein) and mCherry-tagged SNX6. The selected region highlights the dynamic plus-end directed movement of GFP-EB3 for the time period shown as the microtubule grows from the point depicted by the pink arrow to that shown with the green arrow. Each individual frame is then merged (EB3 merge) to show the track and orientation of the microtubule (minus-end at the pink arrow, plus-end at the green arrow). During this period of growth SNX6-labelled endosomes are relatively static. At 1 minute and 25 seconds however, movement of a SNX6-labelled endosome (red arrow) is observed, which tracks stoichastically, at an average speed of approximately 0.35 µm/sec, towards the minus-end of the microtubule (shown by the various time-coded, coloured arrows). The overlay shows the combined images of the individual EB3 merge and SNX6 stills.

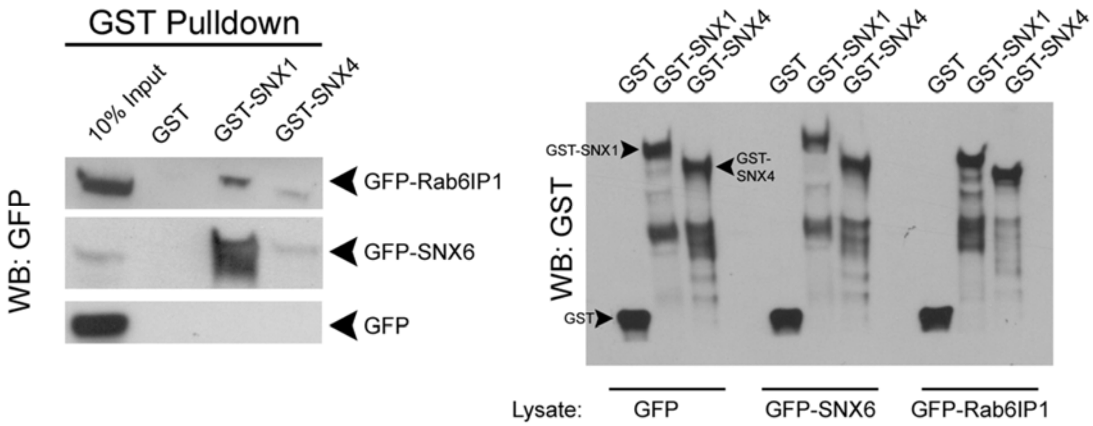

Figure S7. Rab6IP1 Association Is Specific for SNX1 when Compared with SNX4
GST pulldown of bacterially expressed SNX1 and SNX4 demonstrates that GST-SNX1 but not GST-SNX4 can associate with GFP-tagged Rab6IP1.

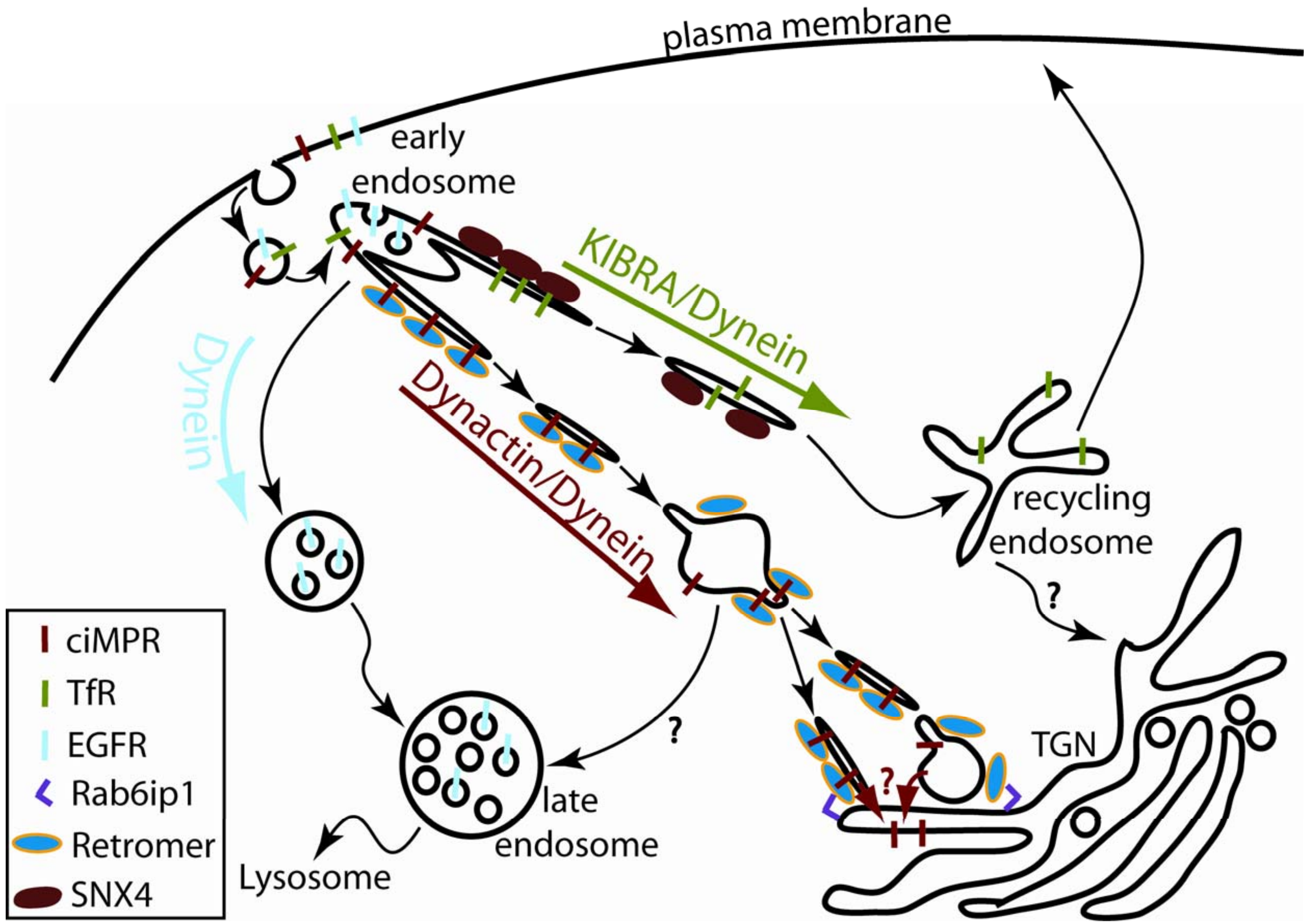

Figure S8. Schematic Representation of s Model for Retromer-Mediated Endosomal Sorting
In this model the involvement of the dynein-dynactin motor in retromer-mediated endosome-to-TGN transport is incorporated, as well as the putative tether Rab6IP1 located at the TGN. Also illustrated are the fusion and fission events of retromer-labelled endosomes visible in live cell imaging. Currently it is not clear what structures actually fuse with the TGN to deliver the CI-MPR (red

arrows indicated with ”?“). Integrated into this model are sorting of the transferrin receptor in a SNX4/KIBRA/dynein dependent pathway[25] and ESCRT mediated sorting of the EGF receptor towards the dynein dependent late endosome/lysosome pathway[44].
