# Supplementary figures and images for: The Retromer Coat Complex Coordinates Endosomal Sorting and Dynein-Mediated Transport, with Carrier Recognition by the trans-Golgi Network
Source: Dev Cell. 2009 Jul 21;17(1):110–22. doi: 10.1016/j.devcel.2009.04.016 (PMC2714578; doi:10.1016/j.devcel.2009.04.016)

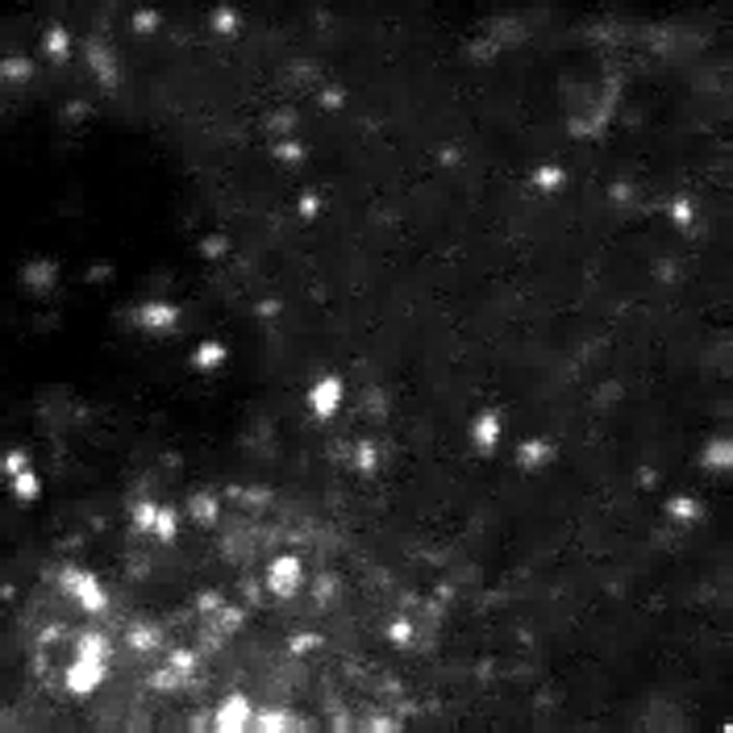

Supplement: Movie S4. Retromer Labeled Vesicles and Tubules Display a Complex Pattern of Movement Involving Numerous Events of Label Merging and Splitting — GFP-SNX6 was lentivirally transfected into HeLa cells and imaged over a 5 min time period (video at ∼17 x real time). The most likely explanation of label merging is fusion of vesicles, while label splitting could be interpreted as fission event. See Figure 4B for scale bar. [file mmc5.jpg]
